# Pharmacology and Therapeutic Potential of Benzothiazole Analogues for Cocaine Use Disorder

**DOI:** 10.1021/acs.jmedchem.3c00734

**Published:** 2023-08-30

**Authors:** Comfort A. Boateng, Ashley N. Nilson, Rebekah Placide, Mimi L. Pham, Franziska M. Jakobs, Noelia Boldizsar, Scot McIntosh, Leia S. Stallings, Ivana V. Korankyi, Shreya Kelshikar, Nisha Shah, Diandra Panasis, Abigail Muccilli, Maria Ladik, Brianna Maslonka, Connor McBride, Moises Ximello Sanchez, Ebrar Akca, Mohammad Alkhatib, Julianna Saez, Catherine Nguyen, Emily Kurtyan, Jacquelyn DePierro, Raymond Crowthers, Dylan Brunt, Alessandro Bonifazi, Amy Hauck Newman, Rana Rais, Barbara S. Slusher, R. Benjamin Free, David R. Sibley, Kent D. Stewart, Chun Wu, Scott E. Hemby, Thomas M. Keck

**Affiliations:** † Department of Basic Pharmaceutical Sciences, Fred Wilson School of Pharmacy, High Point University, One University Parkway, High Point, North Carolina 27268, United States; ‡ Department of Chemistry & Biochemistry, Department of Biological & Biomedical Sciences, College of Science and Mathematics, Rowan University, 201 Mullica Hill Road, Glassboro, New Jersey 08028, United States; § Medicinal Chemistry Section, Molecular Targets and Medications Discovery Branch, National Institute on Drug Abuse-Intramural Research Program, National Institutes of Health, 333 Cassell Drive, Baltimore, Maryland 21224, United States; ∥ Department of Neurology, Johns Hopkins Drug Discovery, The Johns Hopkins University School of Medicine, 855 North Wolfe Street, Baltimore, Maryland 21205, United States; ⊥ Molecular Neuropharmacology Section, National Institute of Neurological Disorders and Stroke-Intramural Research Program, National Institutes of Health, Bethesda, Maryland 20892, United States

## Abstract

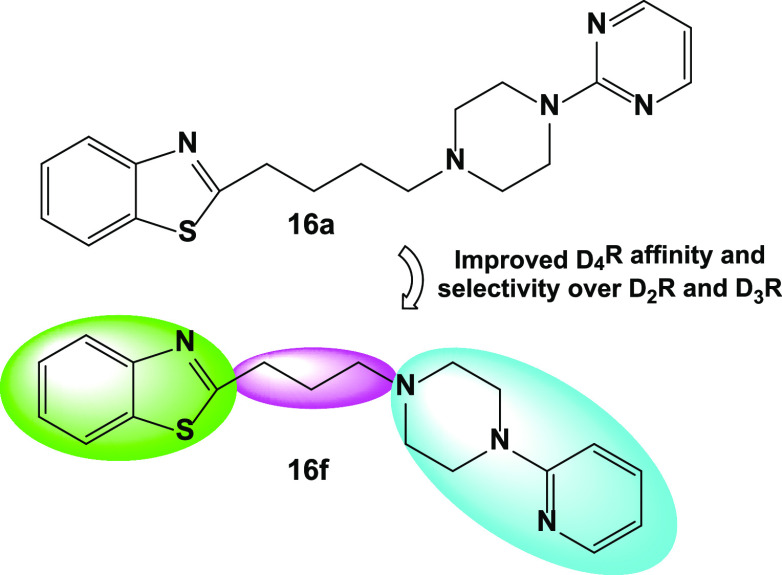

Pharmacological targeting of the dopamine D_4_ receptor (D_4_R)—expressed in brain regions that control cognition, attention, and decision-making—could be useful for several neuropsychiatric disorders including substance use disorders (SUDs). This study focused on the synthesis and evaluation of a novel series of benzothiazole analogues designed to target D_4_R. We identified several compounds with high D_4_R binding affinity (*K_i_* ≤ 6.9 nM) and >91-fold selectivity over other D_2_-like receptors (D_2_R, D_3_R) with diverse partial agonist and antagonist profiles. Novel analogue **16f** is a potent low-efficacy D_4_R partial agonist, metabolically stable in rat and human liver microsomes, and has excellent brain penetration in rats (AUC_brain/plasma_ > 3). **16f** (5–30 mg/kg, i.p.) dose-dependently decreased iv cocaine self-administration in rats, consistent with previous results produced by D_4_R-selective antagonists. Off-target antagonism of 5-HT_2A_ or 5-HT_2B_ may also contribute to these effects. Results with **16f** support further efforts to target D_4_R in SUD treatment.

## Introduction

D_1_-like (D_1_R, D_5_R) and D_2_-like (D_2_R, D_3_R, D_4_R) dopamine receptors are G protein-coupled receptors that regulate physiological functions such as movement, emotion, and cognition.^1,2^ Compared to D_2_R and D_3_R, D_4_Rs have the lowest level of expression in the brain and are uniquely distributed primarily in the prefrontal cortex and hippocampus. In the prefrontal cortex, D_4_R plays important roles in cognition, attention, decision making, and executive function. Common variations in the *DRD4* gene are associated with novelty-seeking, risky behavior, attention-deficit hyperactivity disorder (ADHD) impulse control disorders, and substance use disorders (SUDs).^3−5^ A better understanding of D_4_R-mediated signaling is essential to developing new pharmacotherapeutic treatments.

Studies with D_4_R-selective ligands demonstrate that D_4_R is a promising therapeutic target for the treatment of several neuropsychiatric conditions, notably ADHD and SUDs.^6,7^ D_4_R-selective agonists alter cognition in animal models.^8,9^ In rats, D_4_R-selective agonists A-412997 (**1**) and PD168077 (**2**) improved performance in the novel object recognition task^10,11^ and the five-trial inhibitory avoidance task.^12^

**Figure 1:**
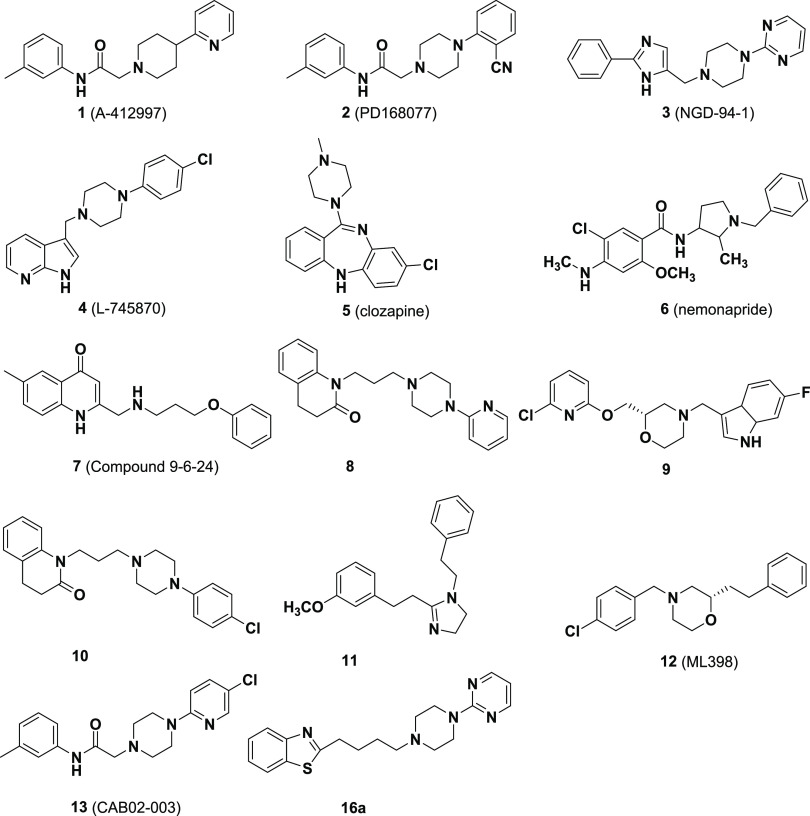
Selected structures of notable D_4_R ligands, including the atypical antipsychotics clozapine (**5**) and nemonapride (**6**). Following the discovery of the relatively high affinity of **5** for D_4_R,^30^ several pharmaceutical companies developed novel ligands targeting D_4_R, including **1**–**4**. A recent resurgence in D_4_R ligand discovery has identified a diverse array of novel agonists (**7**), partial agonists (**8**), and antagonists (**9**–**13**) including lead compound **16a** from this study.

D_4_R antagonism may be useful to treat l-DOPA-induced dyskinesias and SUDs, particularly for psychostimulants like cocaine.^7,13−17^ D_4_R antagonist NGD-94–1 (**3**)^18^ reduced cocaine-maintained behavior in nonhuman primates,^15^ and antagonist L-745,870 (**4**; 3-(4-[4-chlorophenyl]piperazin-1-yl)-methyl-1*H*-pyrrolo[2,3-*b*]pyridine; Figure 1) reduced alcohol-taking and alcohol-seeking behaviors in rats.^19^

One of the most well-studied D_4_R-selective compounds, L-745,870, is >100-fold selective for D_4_R over other dopamine receptors (D_1_R, D_2_R, D_3_R, and D_5_R) with subnanomolar affinity.^20,21^ However, the compound acts as a partial agonist, binds to several nondopaminergic receptors,^22^ and failed to reduce psychotic symptoms in a phase IIa clinical study evaluating its utility as an antipsychotic.^23,24^ These issues are emblematic of many of the research ligands that have been used to investigate D_4_R physiology to date, which stem from unsuccessful attempts in the 1990s to develop new antipsychotics based on the idea that clozapine’s unusually high D_4_R affinity may have been a key driver in its clinical success. The recent resurgence in D_4_R medication development, by our group and many others, has been reviewed ably by others.^6,7^ Several of the intriguing new D_4_R ligands that have been reported recently are shown in Figure 1, including selective, high-affinity agonists (**7**), partial agonists (**8**), and antagonists (**9**–**13**, **16a**).^2,25−29^

Despite the clinical implications of cocaine use disorder (CUD), there are no FDA-approved medications for CUD treatment or approved drugs that selectively target D_4_R. The goal of this study was the development and characterization of novel ligands with high D_4_R affinity and selectivity for investigation in rodent models of CUD. Compound **16a** (2-(4-(4-(pyrimidin-2-yl)piperazin-1-yl)butyl)benzo[*d*]thiazole; Figure 1) was identified in a prior drug development study as a compound with high D_4_R affinity and selectivity over D_2_R and D_3_R, with close analogues having useful effects in treating sexual dysfunction.^31,32^ We used **16a**^31^ as the chemical template for two rounds of structure–activity relationship (SAR)-guided synthesis with the goal of identifying novel druggable D_4_R-selective lead compounds.

We synthesized an initial library of analogues (**16b**–**16f**) featuring modifications to the pyrimidinylpiperazine region of **16a** and variations in the linker chain length. Following extensive *in vitro* analyses, including binding and functional studies, we determined that these modifications resulted in several novel analogues that retained high D_4_R binding affinity, improved D_2_-like subtype selectivity, and showed low-efficacy partial agonist or antagonist profiles at D_4_R. Based on its initial profile, we expanded the characterization of **16f** to include diverse receptor screening, pharmacokinetic and metabolic stability studies, including rat and human liver microsomes, and *in vivo* behavioral analysis in rats trained to self-administer cocaine. In parallel with the *in vivo* characterizations of **16f**, we pursued a second round of SAR studies exploring substitutions on both the terminal benzo[*d*]thiazole and the pyrimidine/pyridine scaffolds as well as piperazine versus piperidine moieties, as identified in an *in silico* screen using the D_4_R crystal structure (crystallized with the antipsychotic nemonapride (**6**; Figure 1) modified via molecular docking.^28^ These diverse libraries provide valuable insight into the SAR driving the D_4_R ligand affinity, efficacy, and target selectivity.

## Chemistry

**Scheme 1:**
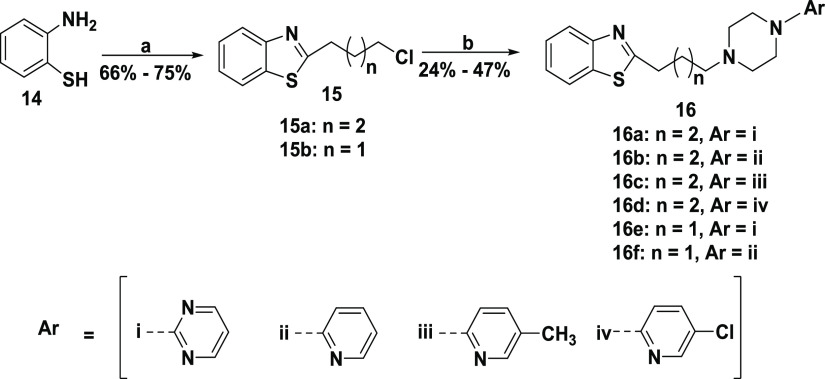
Synthesis of Series 1 of Unsubstituted Benzothiazole Analogues Reagents and conditions: (a) toluene, 5-chloro pentanoyl chloride or 4-chloro butanoyl chloride, RT; (b) CH_3_CN, KI, K_2_CO_3_, reflux, appropriate arylpiperazine or arylpiperidine.

**Scheme 2:**
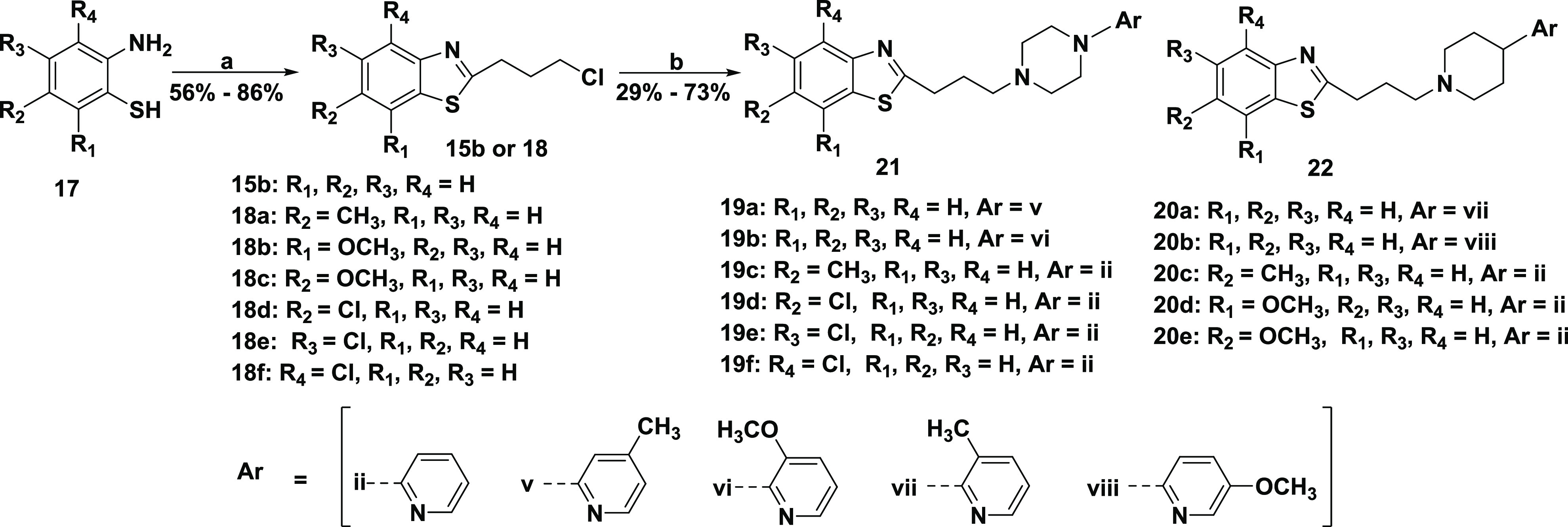
Synthesis of Series 2 of Substituted or Unsubstituted Benzothiazole Analogues Reagents and conditions: (a) toluene, 4-chloro butanoyl chloride, RT; (b) CH_3_CN, KI, K_2_CO_3_, reflux, appropriate arylpiperazine or arylpiperidine.

Our first and second series of ligands were synthesized as outlined in Schemes 1 and 2, respectively, using routine *N*-alkylation reactions previously reported.^2^ In Schemes 1 and 2, substituted or unsubstituted 2-aminobenzenethiol compound **14** or **17** was reacted with 4-chlorobutanoyl chloride or 5-chloropentanoyl chloride to give intermediate compound **15** or **18**. Alkylation of the unsubstituted arylpiperazine moiety with intermediate compound **15** delivered target compound **16**. The same procedure was employed to make target compounds **19** and **20** by alkylation of the substituted or unsubstituted arylpiperazine or arylpiperidine moiety with intermediate compounds **15b** and **18**. The requisite substituted or unsubstituted aminothiophenols and substituted or unsubstituted arylpiperazines or arylpiperidines are commercially available.

In a small initial SAR series, we employed two classes of modifications to parent compound **16a**, varying the alkyl linker chain length and substitutions on the arylpiperazine, producing analogues **16b**–**16f**. In this initial series, the 2-pyrimidine moiety of **16a** was replaced with 2-pyridine in **16b**, 5-methylpyridin-2-yl in **16c**, and 5-chloropyridin-2-yl in **16d**. To evaluate the contribution of the alkyl chain to binding affinity and subtype selectivity, we synthesized alkyl chain length analogues of compounds **16a** and **16b**, removing one methylene from the linker chain in compounds **16e** and **16f**, respectively.

Based on our pharmacological evaluation of this initial series, one compound (**16f**) was chosen for further investigation. Simultaneously, the overall library was later extended with a second series (**19a**–**f**, **20a**–**e**) designed after *in silico* docking studies, as shown in Scheme 2.

This extended library featured additional arylpiperazine/arylpiperidine substitutions as well as modifications to the benzo[*d*]thiazole. In our second series, the pyridine of compound **16f** was substituted with 4-methylpyridin-2-yl to form **19a**. The piperazine attached to the three-C-linker chain on compound **19b** with a 3-methoxy-2-pyridinyl moiety was replaced with a piperidine to form **20a** with a 3-methyl-2-pyridinyl moiety. The 3-methyl-2-pyridine of **20a** was substituted with 5-methoxy-2-pyridine to form **20b**. Finally, we probed the contribution of the benzo[*d*]thiazole moiety via substitution on the phenyl ring with electron-donating (methyl) and electron-withdrawing (chloro) groups (compound **19c** compared to compound **19d**, respectively) with a propyl linker attached to the pyridin-2-yl-piperazin-1-yl moiety. Additional substitutions on the benzo[*d*]thiazole moiety produced compounds 1**9e**,**19f**. We also probed combinations of benzo[*d*]thiazole moiety substitutions with pyridin-2-ylpiperidin-1-yl moieties attached to the propyl linker to obtain compounds **20c**–**20e**.

## Pharmacological Results and Discussion

### SARs at Dopamine D_2_-Like Receptors: Initial Library

Compound **16a** and the structures of all synthesized analogues across both series are shown in Table 1. Overall, all compounds exhibited cLogP values of less than 5 and library members consistently demonstrated higher binding affinity for D_4_R over D_2_R and D_3_R. The binding affinities of all compounds were evaluated via radioligand competition binding studies using [^3^H]*N*-methylspiperone and membranes prepared from HEK293 cells stably expressing human dopamine D_2_-like receptors (D_2_R, D_3_R, or D_4_R). Binding data for all of the ligands are shown in Table 1. In addition, cLogP and polar surface area (PSA) values were calculated to provide measures of polarity (Table 1). Functional analyses of each compound were completed using the LANCE assay for cAMP (Table 2) and the DiscoverX β-arrestin recruitment assay (Table 3), in both agonist and antagonist modes, using Chinese hamster ovary (CHO) cells stably expressing D_2_R, D_3_R, or D_4_R. In agonist mode, *E*_max_ values for each compound are in comparison to dopamine and EC_50_ values represent agonist potency. In antagonist mode, *I*_max_ values for each compound represent inhibition of dopamine-induced signaling and IC_50_ values represent antagonist potency.

**Table 1:**
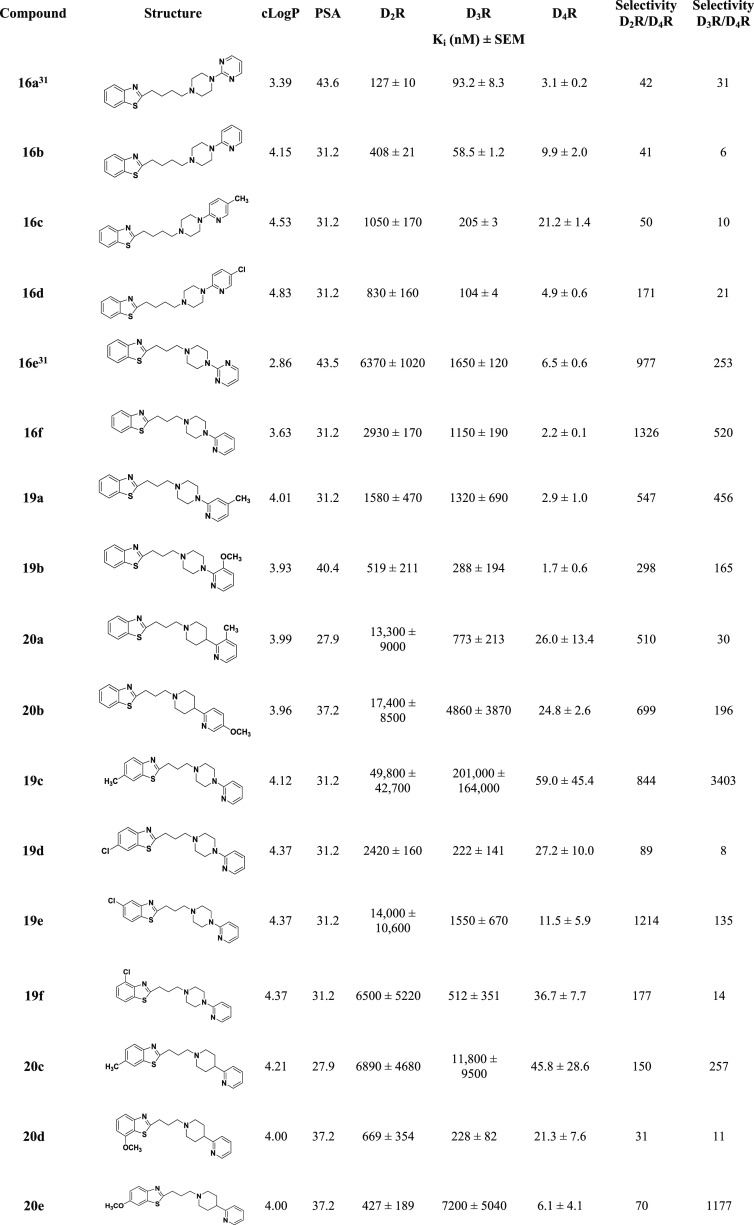
Human Dopamine D_2_-Like Receptor Competition Binding in HEK293 Cells for Benzothiazole Analogues with Varying Three- or Four-Carbon Linker Chains

a *K*_*i*_ values determined by competitive inhibition of [^3^H]*N*-methylspiperone binding in membranes harvested from HEK 293 cells stably expressing hD_2_R, hD_3_R, or hD_4_R. All *K*_*i*_ values are presented as means ± SEM.

**Table 2:** D_2_R- and D_4_R-Mediated Effects on cAMP Production^a^

	**D**_**2**_**R**				**D**_**4**_**R**				**EC**_**50**_	**IC**_**50**_
**compound**	**cAMP***E*_**max**_**%**^b^	**cAMP EC**_**50**_**(nM)**^c^	**cAMP ant. %**^b^	**cAMP IC**_**50**_**(nM)**^c^	**cAMP***E*_**max**_**%**^b^	**cAMP EC**_**50**_**(nM)**^c^	**cAMP ant. %**^b^	**cAMP IC**_**50**_**(nM)**^c^	**selectivity D**_**2**_**R/D**_**4**_**R**	**selectivity D**_**2**_**R/D**_**4**_**R**
dopamine	98.9 ± 2.4	3.46 [2.69–4.44]			101 ± 2	3.78 [3.09–4.63]			0.92	
**spiperone**			102 ± 2	0.59 [0.47–0.73]			104 ± 3	4.91 [3.73–5.86]		
**16a**	inactive	inactive	97.6 ± 5.1	353 [207–605]	inactive	inactive	97.9 ± 2.3	31.8 [24.7–40.9]	ND	11
**16b**	59.4 ± 3.0	124 [68.9–222]	ND	>100,000	inactive	inactive	95.6 ± 2.3	123 [95.9–157]	ND	ND
**16c**	inactive	inactive	107 ± 5	2010 [1390–2910]	inactive	inactive	106 ± 3	600 [467–771]	ND	3.4
**16d**	inactive	inactive	ND	ND	inactive	inactive	ND	10,800 [7520–15,500]	ND	ND
**16e**	80.9 ± 6.9	1180 [577–2390]	ND	>100,000	14.0 ± 0.8	4.34 [1.1–17.1]	82.2 ± 2.0	32.3 [24.7–42.2]	272	ND
**16f**	37.5 ± 3.2	936 [483–1770]	81.3 ± 4.5	6690 [4120–11,000]	14.2 ± 1.2	10.6 [1.6–64.8]	78.8 ± 2.3	69.3 [50.9–94.5]	88	97
**19a**	inactive	inactive	92.8 ± 4.9	628 [375–1040]	inactive	inactive	88.0 ± 4.4	27.8 [15.3–49.2]	ND	23
**19b**	inactive	inactive	86.4 ± 4.4	185 [105–327]	inactive	inactive	90.4 ± 2.6	30.6 [23.1–40.6]	ND	6.0
**20a**	inactive	inactive	104 ± 6	4530 [2740–7500]	inactive	inactive	97.6 ± 4.5	67.2 [41.2–109]	ND	67
**20b**	inactive	inactive	98.3 ± 5.8	2340 [1350–4060]	inactive	inactive	102 ± 5	173 [105–284]	ND	14
**19c**	53.6 ± 4.3	591 [283–1210]	79.4 ± 6.9	3660 [1710–7860]	13.1 ± 2.2	29.5 [1.5–29.3]	77.0 ± 3.5	328 [211–506]	20	11
**19d**	50.2 ± 8.0	1620 [544–4320]	ND	ND	32.3 ± 2.5	272 [110–646]	71.1 ± 4.2	1750 [1020–2990]	6.0	ND
**19e**	78.3 ± 6.0	1800 [1004–3227]	ND	11,600 [5310–25500]	inactive	inactive	73.0 ± 4.0	816 [454–1470]	ND	14
**19f**	84.5 ± 9.7	2050 [871–4760]	62.4 ± 7.8	3040 [629–10800]	inactive	inactive	67.7 ± 5.0	1670 [839–3320]	ND	1.8
**20c**	27.0 ± 3.1	496 [165–1500]	89.9 ± 5.7	719 [380–1340]	24.2 ± 2.1	150 [42.3–593]	77.0 ± 5.7	87.6 [42.6–180]	3.3	8.2
**20d**	42.4 ± 5.1	183 [45.2–649]	92.9 ± 4.7	1840 [1160–2950]	11.3 ± 1.2	12.7 [2.6–58.1]	86.0 ± 4.7	85.5 [46.3–158]	14	22
**20e**	65.3 ± 5.5	228 [102–488]	76.3 ± 5.8	2600 [1190–5490]	30.5 ± 2.1	38.6 [12.8–114]	86.4 ± 4.0	153 [96.3–242]	5.9	17

a Compounds were tested alone (agonist mode) and with an EC_80_ concentration of dopamine (antagonist mode) for their ability to alter cAMP production mediated by D_2_R and D_4_R signaling. Dopamine was used as a control in all agonist mode assays. Spiperone was included in all antagonist mode assays for the D_2_R and D_4_R. ND, not determined due to an incomplete curve. Inactive, no measurable activity.

b Efficacy/antagonist % (ant. %) values obtained from nonlinear regression of meaned data obtained from at least three independent experiments with triplicate measures. Values are presented as means ± SEM.

c Potency values obtained from nonlinear regression of meaned data obtained from at least three independent experiments with triplicate measures. Values are presented as mean [95% confidence interval].

**Table 3:** D_2_R-, D_3_R-, and D_4_R-Mediated β-Arrestin Recruitment.^a^

	**D**_**2**_**R**				**D**_**3**_**R**				**D**_**4**_**R**				**EC**_**50**_		**IC**_**50**_	
**compound**	**β-arr*****E***_**max**_**%**^b^	**β-arr EC**_**50**_**(nM)**^c^	**β-arr ant. %**^b^	**β-arr IC**_**50**_**(nM)**^c^	**β-arr*****E***_**max**_**%**^b^	**β-arr EC**_**50**_**(nM)**^c^	**β-arr ant. %**^b^	**β-arr IC**_**50**_**(nM)**^**b**^	**β-arr*****E***_**max**_**%**^b^	**β-arr EC**_**50**_**(nM)**^c^	**β-arr ant. %**^b^	**β-arr IC**_**50**_**(nM)**^c^	**selectivity D**_**2**_**R/D**_**4**_**R**	**selectivity D**_**3**_**R/D**_**4**_**R**	**selectivity D**_**2**_**R/D**_**4**_**R**	**selectivity D**_**3**_**R/D**_**4**_**R**
dopamine	98.9 ± 1.6	57.1 [47.3–69.0]			100 ± 1	4.27 [3.67–4.96]			95.5 ± 2.0	114 [90.3–145]			0.5	0.04		
spiperone			101 ± 3	1.20 [0.88–1.64]							95.2 ± 2.9	0.80 [0.54–1.18]			1.5	
sulpiride							100 ± 3	69.1 [47.4–99.2]								
**16a**	inactive	inactive	99.0 ± 3.4	242 [167–349]	21.1 ± 1.9	39.4 [10.6–147]	69.0 ± 7.4	1440 [766–2800]	inactive	inactive	100 ± 3	19.1 [13.9–26.4]	ND	ND	13	75
**16b**	32.0 ± 0.9	29.8 [18.6–47.6]	60.2 ± 4.3	849 [450–1600]	82.6 ± 5.3	17.8 [7.34–43.1]	inactive	inactive	inactive	inactive	97.8 ± 3.6	104 [71.1–153]	ND	ND	8.2	ND
**16c**	inactive	inactive	104 ± 4	1560 [1120–2170]	inactive	inactive	101 ± 8	937 [541–1630]	inactive	inactive	108 ± 4	275 [185–408]	ND	ND	5.7	3.4
**16d**	inactive	inactive	108 ± 19	23,000 [8960–67,700]	inactive	inactive	ND	ND	inactive	inactive	103 ± 4	414 [274–621]	ND	ND	56	ND
**16e**	29.9 ± 0.9	1060 [769–1470]	58.5 ± 6.5	10,200 [4820–22,500]	58.4 ± 1.8	2340 [1680–3260]	50.6 ± 15	15,100 [4010–65,800]	inactive	inactive	108 ± 4	71.5 [48.8–105]	ND	ND	143	211
**16f**	12.5 ± 0.5	1110 [710–1740]	94.6 ± 6.9	10,000 [5890–17,200]	47.7 ± 2.6	5560 [3440–8990]	56.4 ± 10	22,000 [9050–60,200]	inactive	inactive	105 ± 4	25.6 [17.5–37.4]	ND	ND	391	859
**19a**	inactive	inactive	102 ± 6	6430 [4010–10,300]	inactive	inactive	101 ± 10	12,900 [7490–22,400]	inactive	inactive	88.6 ± 4.6	6.2 [2.6–17.6]	ND	ND	1040	2087
**19b**	inactive	inactive	94.0 ± 3.6	414 [268–633]	inactive	inactive	81.5 ± 3.4	474 [298–747]	inactive	inactive	91.7 ± 3.6	2.2 [1.4–3.4]	ND	ND	191	218
**20a**	inactive	inactive	100 ± 2	9920 [7660–12,900]	inactive	inactive	101 ± 2	13,300 [9450–18,700]	inactive	inactive	100 ± 2	52.4 [40.3–68.3]	ND	ND	189	254
**20b**	inactive	inactive	107 ± 5.2	14,300 [10,500–19,600]	inactive	inactive	112 ± 7	8460 [5590–12,900]	inactive	inactive	100 ± 3	333 [244–454]	ND	ND	43	25
**19c**	37.1 ± 4.8	18,900 [6600–56,300]	89.6 ± 7.5	15,600 [9330–26,400]	60.5 ± 6.3	13100 [4130–38700]	64.9 ± 11	15,300 [6270–41,600]	8.9 ± 1.2	8.81 [1.15–112]	100 ± 6	2340 [1370–3950]	2148	1481	6.7	6.5
**19d**	ND	ND	75.2 ± 12	24,200 [9680–65,800]	ND	ND	inactive	inactive	inactive	inactive	93.9 ± 5.4	3260 [1970–5310]	ND	ND	7.4	ND
**19e**	ND	ND	ND	ND	ND	ND	32.8 ± 7.2	24,000 [9070–76,600]	inactive	inactive	104 ± 5	377 [221–631]	ND	ND	ND	64
**19f**	ND	ND	ND	ND	ND	ND	inactive	inactive	inactive	inactive	102 ± 6	2290 [1330–3860]	ND	ND	ND	ND
**20c**	inactive	inactive	92.5 ± 3.8	2780 [1980–3910]	inactive	inactive	97.0 ± 6.3	6700 [4330–10,400]	inactive	inactive	98.6 ± 2.7	248 [187–237]	ND	ND	11	27
**20d**	inactive	inactive	98.3 ± 3.4	2260 [1660–3070]	inactive	inactive	107 ± 6	2770 [1820–4200]	inactive	inactive	103 ± 3	122 [86.5–172]	ND	ND	19	23
**20e**	29.0 ± 2.0	1170 [498–2890]	64.9 ± 3.7	4460 [2910–6810]	31.4 ± 2.5	3790 [1400–9660]	83.7 ± 8.6	8780 [4630–17,100]	inactive	inactive	93.7 ± 3.0	724 [528–990]	ND	ND	6.2	12

a Compounds were tested alone (agonist mode) and with an EC_80_ concentration of dopamine (antagonist mode) for their ability to alter β-arrestin recruitment to D_2_R, D_3_R, and D_4_R. Dopamine was used as a control in all agonist mode assays. Spiperone was included in all antagonist mode assays for D_2_R and D_4_R. Sulpiride was included in all antagonist mode assays with the D_3_R. ND, not determined due to an incomplete curve. Inactive, no measurable activity.

b Efficacy/antagonist % (ant. %) values obtained from nonlinear regression of meaned data obtained from at least three independent experiments with triplicate measures. Values are presented as means ± SEM.

c Potency values obtained from nonlinear regression of meaned data obtained from at least three independent experiments with triplicate measures. Values are presented as mean [95% confidence interval].

In our initial series, modification of the pyrimidine ring of **16a** to a pyridine or substituted pyridine (**16b**–**16d**) modestly decreased D_4_R binding affinity in competition binding assays and decreased fold selectivity over the structurally related D_3_R. Compounds **16b** and **16c** had decreased but still highly potent D_4_R binding affinity at 9.9 ± 2.0 and 21.2 nM ± 1.37, respectively. The D_2_R/D_4_R fold selectivity was similar to compound **16a** at ∼40-fold, but the D_3_R/D_4_R fold selectivity decreased to 6-fold (**16b**) and 10-fold (**16c**). In cAMP antagonism assays, **16b** and **16c** were full antagonists (∼100% inhibition) at D_4_R but lost potency (IC_50_ = 123 nM [95.9–157] and 600 nM [467–771], respectively) compared to **16a** (IC_50_ = 31.8 nM [24.7–40.9]). Furthermore, **16b** inhibited cAMP production via activation of D_2_R (*E*_max_ = 59.4 ± 3.0%, EC_50_ = 124 nM [68.9–222]) while the methyl substitution in **16c** remained inactive in agonist mode but was a full D_2_R antagonist with low potency (*I*_max_ = 107 ± 5, IC_50_ = 2010 [1390–2910]). Similar results for D_4_R and D_2_R were seen in the β-arrestin assay. **16b** was a full antagonist at D_4_R (*I*_max_ = 97.8 ± 3.6%, IC_50_ = 104 nM [71.7–153]) but was a partial agonist at D_2_R (*E*_max_ = 32.0 ± 0.9%, EC_50_ = 29.8 nM [18.6–47.6]). Additionally, **16b** was a potent agonist at D_3_R for β-arrestin recruitment (*E*_max_ = 82.6 ± 5.3, EC_50_ = 17.8 nM [7.34–43.1]. As with the cAMP assay, the addition of the methyl substituent in **16c** removed any agonist activity in the β-arrestin recruitment assay. **16c** was a full antagonist at D_2_R (*I*_max_ = 104 ± 4%, IC_50_ = 1560 nM [1120–2170]), D_3_R (*I*_max_ = 101 ± 8%, IC_50_ = 937 nM [541–1630]), and D_4_R (*I*_max_ = 108 ± 4%, IC_50_ = 275 nM [185–408]) but did not show high selectivity among the receptors (<6-fold). Replacing the pyrimidine ring with a pyridine ring introduced D_2_R and D_3_R partial agonist activity in β-arrestin recruitment assays, while adding a methyl substituent to the pyridine ring restored full antagonism at all receptors.

The chloro-substituted pyridine **16d** maintained a high D_4_R binding affinity (*K_i_* = 4.9 ± 0.6 nM) with decreased D_2_R affinity (*K_i_* = 830 ± 160 nM) compared to **16a** (*K_i_* = 127 ± 10 nM), which improved D_4_R selectivity over D_2_R 171-fold. **16d** maintained a D_3_R binding affinity similar to that of **16a** and thus had no improvement in D_4_R selectivity over D_3_R. However, cAMP and β-arrestin recruitment were greatly diminished across all receptors. Starting with D_4_R, **16d** lost potency in cAMP assays with the estimated IC_50_ = 10,800 nM [7520–15,500]. In the β-arrestin assay, **16d** was a full antagonist (*I*_max_ = 103 ± 4%, IC_50_ = 414 nM [274–621]). At D_2_R, **16d** had greatly reduced potency at both cAMP (*I*_max_ and IC_50_ = not determined) and β-arrestin recruitment (*I*_max_ = 108 ± 19%, IC_50_ = 23,000 nM [8960–67,700]). **16d** did not recruit β-arrestin to D_3_R, indicating no detectable agonist activity, and very low potency antagonism was suggested but accurate potency was not determined due to incomplete (unsaturated) curves. Although the D_4_R binding affinity was not affected by the chloro substituent, functional activity was greatly diminished for all D_2_-like receptors.

Removing one methylene unit from the linker chain—decreasing the alkyl linker from four carbons to three—markedly improved selectivity for D_4_R by reducing D_2_R and D_3_R binding affinities. Compared to similar butyl linker compounds **16a**–**16c**, the propyl linker chain in **16e**,**f**and **19a** maintained the D_4_R binding affinity and greatly reduced the D_2_R and D_3_R binding affinity. For example, **16e** had a D_4_R binding affinity (*K_i_* = 6.5 ± 0.6 nM) similar to that of **16a** (*K_i_* = 3.1 ± 0.2 nM) but a dramatically decreased affinity for D_2_R (**16e**: *K_i_* = 6370 ± 1020 nM; **16a**: *K_i_* = 127 ± 10 nM) and D_3_R (**16e**: *K_i_* = 1650 ± 120 nM; **16a**: *K_i_* = 93.2 ± 8.3 nM). Exchanging the pyrimidine in **16e** for a pyridine in **16f** improved D_4_R binding affinity (*K_i_* = 2.2 ± 0.1 nM) and further increased D_4_R selectivity over both D_2_R (1326-fold) and D_3_R (520-fold). In cAMP functional assays, **16e** (*E*_max_ = 14.0 ± 0.8%, EC_50_ = 4.3 nM [1.1–17.1]) and **16f** (*E*_max_ = 14.2 ± 1.2%, EC_50_ = 10.6 nM [1.6–64.8]) exhibited low partial agonism at D_4_R. Corresponding antagonist mode assays for **16e** (*I*_max_ = 82.2 ± 2.0%, IC_50_ = 32.3 nM [24.7–42.2]) and **16f** (*I*_max_ = 78.8 ± 2.3%, IC_50_ = 69.3 nM [50.9–94.5]) indicated they were partial antagonists. Higher efficacy but much lower potency for **16e** and **16f** were observed at D_2_R in both the agonist and antagonist modes. The potency of **16e** at D_2_R was >100,000 nM in antagonist mode. In agonist mode at D_2_R, **16e** was a high-efficacy partial agonist (*E*_max_ = 80.9 ± 6.9%, EC_50_ = 1180 nM [577–2390]) with D_4_R selectivity (272-fold). The D_4_R selectivity of **16f** was 97-fold over D_2_R in antagonist mode due to decreased D_2_R potency (*I*_max_ = 81.3 ± 4.5%, IC_50_ = 6690 nM [4120–11,000]). In the β-arrestin recruitment assays, **16e** and **16f** were full antagonists at D_4_R with potencies of 71.5 and 25.6 nM, respectively. At D_2_R and D_3_R, **16e** and **16f** were partial agonists/antagonists with potencies >1000 nM. Of note, **16f** had excellent selectivity over D_2_R (391-fold) and D_3_R (859-fold).

### *In Silico* Screening to Guide SAR Library Expansion with a Second Series

#### Combinatorial Library of Compound **16f**

Structural variations of the initial lead compound **16f** template were generated, producing a permutational library of 4400 D_4_R ligands in total, as shown in Figure S1. Individual libraries were built for each core variance shown with simple substitutions using the Combinatorial Library Enumeration tool in Maestro software (Schrödinger): scaffold 1:6 × 6 × 6 × 6 (1296); scaffold 2:4 × 4 × 4 × 4 × 2(512); scaffold 3:6 × 6 × 6 × 6 × 2 (2592).

#### Preparation of the Protein and Ligand Library

The crystal structure (5WIU) of D4R in complex with nemonapride **2** (chemical structure shown in Figure 1) was prepared and preprocessed using Maestro’s Protein Preparation Wizard.^33^ The preprocessed protein’s charge state was optimized at pH 7.4. Then, a restrained minimization was performed to relax the protein structure using the OPLS3 force field.^34^ Using Maestro Elements allowed for the preparation of the 3D structures of nemonapride **2** and the 4400 **16f** analogues. The 3D structure of nemonapride was extracted from the crystal structure (PDB ID: 5WIU), and the initial structure of the **16f** analogues was from the Combinatorial Library Enumeration tool. In order to generate each ligand’s ionization/tautomeric states at pH 7.4, Maestro’s Epik tool was used based on the more accurate Hammett and Taft methodologies.^33^ During this step, the lowest ionization/tautomeric state was chosen. Afterward, the geometry was minimized to the most energetically favorable structure to relax the ligand’s structure.

#### Glide XP Docking and Compound Selection

The receptor grid files were generated from the prepared receptor complex in which the centroid of the crystal ligand, nemonapride, was used to specify the active site. The prepared ligands were docked into their corresponding generated grids using Glide XP scoring with default procedures and parameters.^34^ In detail, the receptor grid required for the docking process was generated using a van der Waals scaling factor of 1 and a partial charge cutoff of 0.25. Docking was performed by using a ligand-centered grid and an OPLS3 force field. Glide XP Dock was used to perform a comprehensive systematic search for the best receptor conformations and orientations to fit the ligand. The docked pose of the crystal ligand was confirmed with its crystal pose, thus validating the docking protocol. Figure S2 shows the interactions between **16f** and residues within the D_4_R binding pocket.

Following Glide XP docking, we selected several compounds per library, focusing on compounds with the simplest substitutions while still maintaining improved docking scores relative to those of the lead scaffold. Table S2 represents the 10 identified ligands with desirable docking scores and with the greatest synthetic feasibility. Both interaction diagrams (left) and 3D representations with interacting residues (right) are provided for the best selected candidate in each of our list.

### SARs at Dopamine D_2_-Like Receptors: Expanded Library

Adding a 4-methyl substituent onto the pyridine ring (**19a**) maintained high D_4_R affinity (*K_i_* = 2.9 ± 1.0 nM) and subtype selectivity of >450-fold over both D_2_R and D_3_R, with no agonist activity detected for any receptor in either cAMP or β-arrestin recruitment assays. At D_4_R, **19a** potently antagonized cAMP inhibition (*I*_max_ = 88.0 ± 4.4%, IC_50_ = 27.8 nM [15.3–49.2]) and β-arrestin recruitment (*I*_max_ = 88.6 ± 4.6%, IC_50_ = 6.2 nM [2.6–17.6]). **19a** antagonized D_2_R-mediated cAMP inhibition (*I*_max_ = 92.8 ± 4.9%, IC_50_ = 628 nM [375–1040]) but was less potent at inhibiting β-arrestin recruitment to D_2_R (*I*_max_ = 102 ± 6%, IC_50_ = 6430 nM [4010–10,300]) and D_3_R (*I*_max_ = 101 ± 10%, IC_50_ = 12900 nM [7490–22,400]) compared to its potency at D_4_R.

Compared to **16f**, the addition of a 3-methoxy substitution on the pyridine ring (**19b**) improved binding affinity across D2-like receptors (D_4_R *K_i_* = 1.74 ± 0.58 nM; D_2_R *K_i_* = 519 ± 211 nM; D_3_R *K_i_* = 288 ± 194 nM), resulting in a small loss of D_4_R-subtype selectivity. In cAMP antagonist assays, **19b** gained potency at D_2_R (*I*_max_ = 86.4 ± 4.4, IC_50_ = 185 nM [105–327]) compared to **16f**, resulting in decreased D_4_R selectivity at sixfold for cAMP assays. The potency of **19b** for D_4_R-mediated cAMP inhibition antagonism was 30.6 nM (*I*_max_ = 90.4 ± 2.6, IC_50_ = 30.6 nM [23.1–40.6]). **19b** was inactive as an agonist at both D_2_R and D_4_R. **19b** was also inactive for all receptors tested in agonist mode for β-arrestin recruitment inhibition but was highly potent at D_4_R in antagonist mode (*I*_max_ = 91.7 ± 3.6%, IC_50_ = 2.2 nM [1.4–3.4]). **19b** was much less potent for β-arrestin recruitment inhibition at D_2_R (*I*_max_ = 94.0 ± 3.6%, IC_50_ = 414 nM [268–633]) and D_3_R (*I*_max_ = 81.5 ± 3.4%, IC_50_ = 474 nM [298–747]). Together, these results indicate the importance of the propyl linker length in D_4_R affinity and subtype selectivity. Furthermore, substitutions on the pyridin-2-yl-piperazin-1-yl moieties can dramatically alter the intrinsic efficacy of each compound.

We probed the importance of the piperazine ring by replacing it with a piperidine in compounds **20a** and **20b**. While we lack matching piperazine analogues, comparing **20a** and **20b** to the **16a**–**f** and **19a**,**b** series shows a loss of D_4_R binding affinity (*K_i_* = 24–26 nM) but excellent selectivity over D_2_R (>500-fold) due to a marked reduction in D_2_R binding affinity. In functional assays, **20a** was a full antagonist at D_4_R (cAMP: *I*_max_ = 97.6 ± 4.5%, IC_50_ = 67.2 [41.2–109]; β-arrestin: *I*_max_ = 100 ± 2%, IC_50_ = 52.4 nM [40.3–68.3]) and had lower potency at D_2_R (cAMP: *I*_max_ = 104 ± 6%, IC_50_ = 4530 nM [2740–7500]; β-arrestin: *I*_max_ = 100 ± 2%, IC_50_ = 9920 nM [7660–12,900]) and D_3_R (β-arrestin: *I*_max_ = 101 ± 2%, IC_50_ = 13,300 nM [9450–18,700]). **20b** was an antagonist in cAMP and β-arrestin recruitment assays for all receptors tested but did not maintain selectivity for D_4_R over D_2_R compared to **20a** (cAMP: 14-fold; β-arrestin: 43-fold) or D_3_R (β-arrestin: 25-fold selective).

The benzo[*d*]thiazole moiety represents a new secondary pharmacophore for the arylpiperazine/arylpiperidine class of D_2_-like ligands. We evaluated the suitability of modifying this region of the molecule by adding electron-donating and electron-withdrawing groups onto the phenyl ring, producing mixed effects on binding affinity. 6-Methylbenzo[*d*]thiazole (**19c**) and 6-chlorobenzo[*d*]thiazole (**19d**) both reduced D_4_R binding affinity (*K_i_* = 59.0 ± 45.4 nM and *K_i_* = 27.2 ± 10.0 nM, respectively) compared to the unsubstituted analogue **16f** (*K_i_* = 2.2 ± 0.1 nM). While the electron-donating methyl group on **19c** produced excellent selectivity over D_2_R (844-fold) and D_3_R (3403-fold), an electron-withdrawing chloro substituent (**19d**) resulted in increased D_2_R and D_3_R affinity and greatly reduced D_4_R selectivity compared to **19c** (89- and 8-fold, respectively). **19c** and **19d** had similar D_4_R functional profiles: partial agonists for receptor-mediated cAMP inhibition (**19c**: *E*_max_ = 13.1 ± 2.2%, EC_50_ = 29.5 nM [1.5–29.3]; **19d**: *E*_max_ = 32.3 ± 2.5%, EC_50_ = 272 nM [110–646]) but antagonists for β-arrestin recruitment (**19c**: *I*_max_ = 100 ± 6%, IC_50_ = 2340 nM [1370–3950]; **19d**: *I*_max_ = 93.9 ± 5.4%, IC_50_ = 3260 nM [1970–5310]. At D_2_R and D_3_R, **19c** and **19d** exhibited similar partial agonism for cAMP (*E*_max_ = ∼ 50–54%) but diverged in β-arrestin efficacy, where **19c** was a partial agonist (*E*_max_ = 37.1 ± 4.8% at D_2_R, *E*_max_ = 60.5 ± 6.3% at D_3_R) and **19d** had no measurable β-arrestin recruitment, suggesting that electron-donating/withdrawing groups at this position can substantially alter receptor signaling characteristics.

Comparing 6-chlorobenzo[*d*]thiazole analogue **19d** to 5-chlorobenzo[*d*]thiazole analogue **19e** and 4-chlorobenzo[*d*]thiazole analogue **19f** reveals other marked effects. These variations decreased the D_4_R binding affinity (*K_i_* = ∼ 11–37 nM) but concomitantly decreased the D_2_R and D_3_R binding affinities as well, resulting in improved D_4_R selectivity. **19d** is a D_4_R partial agonist at cAMP (*E*_max_ = 32.3%), but **19e** and **19f** have no detectable D_4_R agonist activity and are full antagonists; all are full antagonists for β-arrestin recruitment. At D_2_R, **19e** and **19f** exhibited higher partial D_2_R agonism for cAMP (*E*_max_ = ∼ 78–85%) than **19d** (*E*_max_ = 50.2%) but had little-to-no detectable β-arrestin recruitment. At D_3_R, **19d** and **19f** have no detectable β-arrestin recruitment but the 5-chloro analogue **19e** gains partial agonism (*E*_max_ = 32.8 ± 7.2%) albeit at very low potency (24,000 nM [9070–76,600]). Together, these data indicate that methyl and chloro substituents on benzo[*d*]thiazole can shift the functional profile at D_4_R with a moderate loss of potency. However, the decreased D_4_R potency appears to be less sensitive to these substituents than the D_2_R and D_3_R substituents, where affinity and functional potency are nearly ablated.

Replacing the 1-(pyridin-2-yl)piperazine of **19c** with 2-(piperidin-4-yl)pyridine (**20c**) resulted in a slight improvement in the binding affinity across all receptors. However, D_4_R selectivity over D_2_R (150-fold) and D_3_R (257-fold) decreased compared to **19c**. **20c** had partial agonist efficacy at D_4_R-mediated cAMP inhibition (*E*_max_ = 24.2 ± 2.1%, EC_50_ = 150 nM [42.3–593]) and D_2_R-mediated cAMP inhibition (*E*_max_ = 27.0 ± 3.1%, EC_50_ = 496 nM [165–1500]). In β-arrestin recruitment assays, **20c** was inactive as an agonist for all receptors tested and a full antagonist at D_4_R (*I*_max_ = 98.6 ± 2.7%, IC_50_ = 248 nM [187–237]), D_2_R (*I*_max_ = 92.5 ± 3.8%, IC_50_ = 2780 nM [1980–3910]), and D_3_R (*I*_max_ = 97.0 ± 6.3%, IC_50_ = 6700 nM [4330–10,400]).

Replacing the methyl group with a methoxy group at the 4 position (**20e**) and then moving the methoxy group to the 3 position (**20d**) improved D_4_R binding affinity (*K_i_* = 6.1 ± 4.1 nM and *K_i_* = 21.3 ± 7.6 nM, respectively) compared to **20c**. However, **20d** gained D_2_R and D_3_R affinity (*K_i_* = 669 ± 354, *K_i_* = 228 ± 82 nM, respectively) while **20e** only gained D_2_R binding affinity (*K_i_* = 427 ± 189 nM). **20d** was a cAMP partial agonist at D_4_R (*E*_max_ = 11.3 ± 1.2%, EC_50_ = 12.7 nM [2.6–58.1]) and D_2_R (*E*_max_ = 42.4 ± 5.1%, EC_50_ = 183 nM [45.2–649]), and it exhibited antagonism for β-arrestin recruitment at all receptors (D_2_R, *I*_max_ = 98.3 ± 3.4%, IC_50_ = 2260 nM [1660–3070]; D_3_R, *I*_max_ = 107 ± 6%, IC_50_ = 2770 nM [1820–4200]; D_4_R, *I*_max_ = 103 ± 3%, IC_50_ = 122 nM [86.5–172]). **20e** was also a partial agonist at D_4_R (*E*_max_ = 30.5 ± 2.1%, EC_50_ = 38.6 nM [12.8–114]) and D_2_R (*E*_max_ = 65.3 ± 5.5%, EC_50_ = 228 nM [102–488]) for cAMP inhibition. In notable contrast to **20d**, **20e** exhibited partial β-arrestin recruitment agonism at D_2_R (*E*_max_ = 29.0 ± 2.0%, EC_50_ = 1170 nM [498–2890]) and D_3_R (*E*_max_ = 31.4 ± 2.5%, EC_50_ = 3790 nM [1400–9660]) but no agonist activity was detected at D_4_R where it was an antagonist (*I*_max_ = 93.7 ± 3.0%, IC_50_ = 724 nM [528–990]).

Overall, the initial and expanded library included four key classes of modifications with distinct effects on binding and efficacy profiles across the D_2_-like receptors. We find the following SARs: (1) reducing the linker chain length from a butyl linker to a propyl linker dramatically improved D_4_R binding selectivity over D_2_R and D_3_R. This is consistent with prior literature^25,35,36^ that supports alkyl linker length substantially driving D_2_-like subtype selectivity, with a propyl linker providing optimal separation of the lipophilic and basic moieties to improve D_4_R affinity and selectivity. (2) Substitution of the pyrimidine ring in initial lead **16a** with a pyridinyl moiety further improved D_4_R binding affinity and selectivity over D_2_R and D_3_R. (3) Piperazine and piperidine ring moieties produce differential effects on cAMP and β-arrestin signaling at each receptor. (4) Substitutions at different positions on the benzo[*d*]thiazole moiety substantially altered binding and functional profiles and warranted more detailed follow-up studies. We also note that 5-substituted pyridine rings (**16c**, **16d**, **20b**) were full antagonists, consistent with prior published reports.^2,37,38^

**Figure 2:**
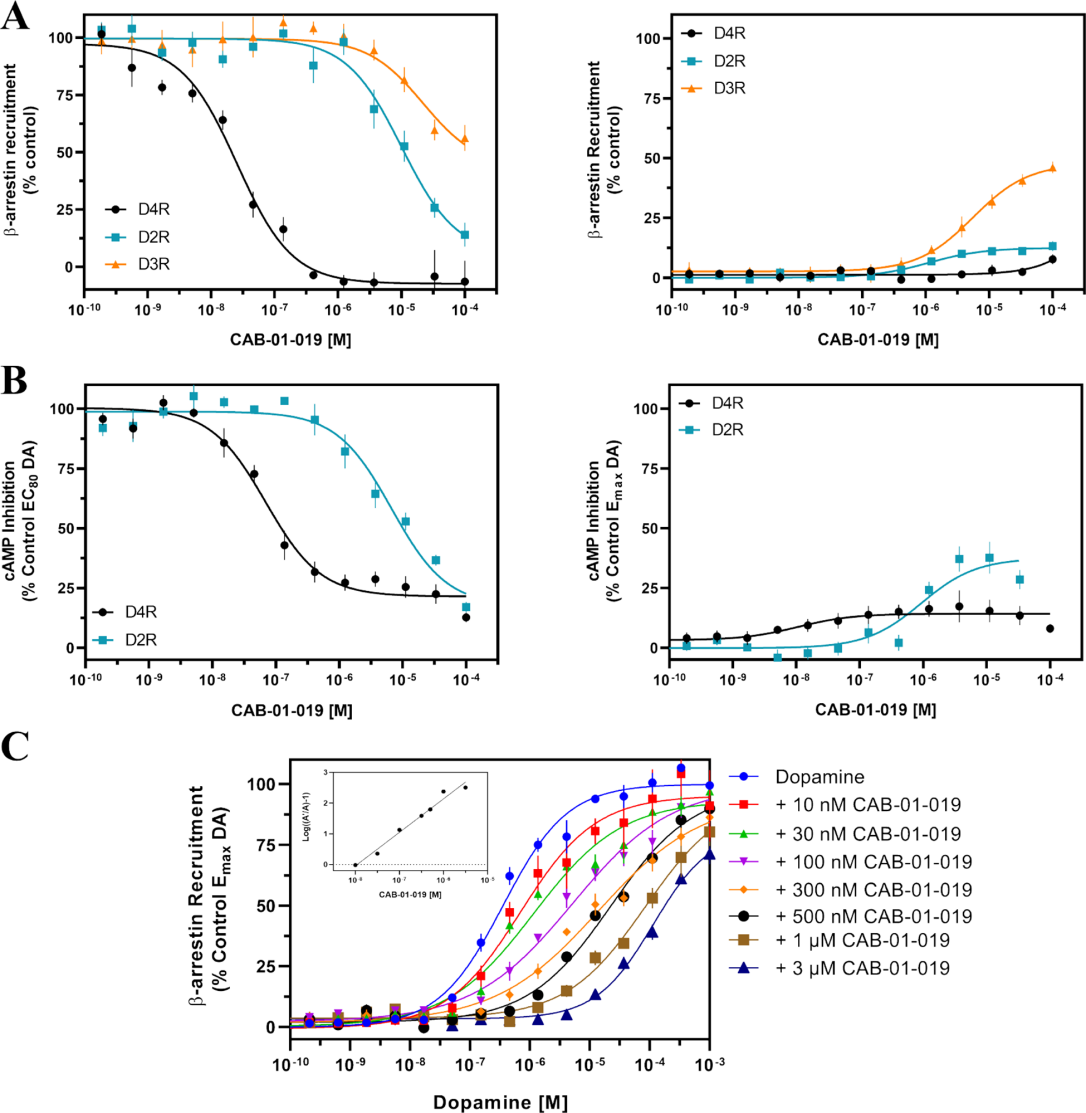
Lead compound **16f** (**CAB-01–019**) demonstrated excellent D_4_R selectivity in functional assays and is a competitive antagonist at D_4_R. (A) **16f** is a potent full D_4_R antagonist for β-arrestin recruitment with no D_4_R agonist activity detected. At D_2_R and D_3_R, **16f** is a low-potency antagonist that shows 391- and 859-fold selectivity for the D_4_R, respectively (Table 3). D_3_R exhibits partial agonist activity with **16f**, while D_2_R has very low partial agonist activity. (B) **16f** potently antagonizes D_4_R-mediated cAMP inhibition and is 97-fold more potent at D_4_R than at D_2_R (Table 2). Furthermore, **16f** has low-efficacy D_4_R agonism and is a low-potency partial agonist at D_2_R. (C) With increasing concentrations of **16f**, dopamine concentration–response curves are shifted to the right with no decrease in *E*_max_, indicating that **16f** is a competitive orthosteric ligand. Furthermore, the Schild plot (inset) of these data had a slope of 1.09 and *K*_b_ = 11.0 nM. All data are presented as means ± SEM from at least three independent experiments run in triplicate.

**16f** was chosen for further analysis based on its pharmacological profile: high D_4_R binding affinity with excellent selectivity over D_2_R and D_3_R (1326**-** and 520-fold, respectively), as measured by [^3^H]*N*-methylspiperone competition (Table 1), and excellent D_4_R selectivity in both cAMP and β-arrestin recruitment antagonism (Tables 2 and 3). It was also one of our first compounds to complete *in vitro* characterization. **16f** is a low-efficacy D_4_R partial agonist, as measured in cAMP inhibition assays (Figure 2A and Table 2), and a full antagonist in β-arrestin recruitment assays (Figure 2B and Table 3), maintaining 97-fold D_4_R selectivity over D_2_R in cAMP antagonist assays, 391-fold D_4_R selectivity over D_2_R in β-arrestin recruitment antagonist assays, and 859-fold D_4_R selectivity over D_3_R in β-arrestin recruitment antagonist assays, indicating it is highly subtype-selective. We conducted Schild-type analysis of **16f** using a β-arrestin recruitment assay to determine whether **16f** was a competitive orthosteric antagonist without any allosteric activity. Dopamine concentration–response curves were conducted in the presence of DMSO and with increasing concentrations of **16f** (Figure 2C). The dopamine curves shifted to the right without decreasing dopamine efficacy, indicating that **16f** is a competitive antagonist. Schild-type analysis revealed the slope-approached unity (slope = 1.09), and its affinity was 11.0 nM (Figure 2C inset). Together, these results indicated that **16f** is a potent and selective D_4_R antagonist suitable for further analyses.

### Compound **16f** Selectivity at an Array of CNS GPCRs and Monoamine Transporters

Our results indicated that **16f** is highly D_4_R-selective compared to the other D_2_-like receptors (D_2_R and D_3_R). In order to determine the selectivity of **16f** at other biogenic amine receptors, **16f** was tested at the Psychoactive Drug Screening Program (PDSP), which tests compounds at an array of GPCRs and monoamine transporters.^39^ In an initial high-concentration (10 μM) screen, **16f** displayed greater than 50% radioligand inhibition at 23 GPCRs/transporters. These were further tested in full concentration–response analyses to determine the affinity of **16f** for each receptor/transporter (Table 4). Only six GPCRs showed an affinity higher than 100 nM: σ_2_, 5-HT_1A_, 5-HT_2A_, 5-HT_2B_, α_2C_, and D_4_R (Table 4). The PDSP-determined affinity of **16f** for D_4_R was 9.48 nM, consistent with the results we obtained (*K_i_* = 2.21 nM). **16f** had comparable affinity for 5-HT_1A_ (5.8 nM) and 5-HT_2B_ (13 nM) and lower affinity for 5-HT_2A_ (46 nM), α_2C_ (73 nM), and σ_2_ (73 nM; Table 4). Given the important roles that serotonin receptors can play in SUDs, we further characterized the signaling effects of **16f** at the three highest affinity secondary targets, 5-HT_1A_, 5-HT_2A_, and 5-HT_2B_.

**Table 4:** Psychoactive Drug Screening Program (PDSP) Results from Primary and Secondary Assays on an Array of Receptors and Monoamine Transporters^a^

**receptor/transporter**	**primary screen** (% inhibition)	**secondary assay *K*_*i*_ (nM)**^b^
D1	59	2736
D2	39	NT
D3	72	584
**D4**	78	**9.48**
D5	–0.04	NT
5-HT1A	109	**5.80**
5-HT1B	68	2859
5-HT1D	75	1423
5-HT1E	60	2764
5-HT2A	98	**46.0**
5-HT2B	99	**13.0**
5-HT2C	73	3545
5-HT3	38	NT
5-HT5A	56	2266
5-HT6	3	NT
5-HT7	90	107
MOR	60	1265
DOR	8	NT
KOR	19	NT
Alpha1A	89	586
Alpha1B	89	397
Alpha1D	88	4262
Alpha2A	85	356
Alpha2B	85	358
**Alpha2C**	94	**72.0**
H1	85	431
H2	13	NT
H3	18	NT
H4	13	NT
Sigma 1 GP	85	252
Sigma 2	84	**72.7**
GABAA	35	NT
Beta1	32	NT
Beta2	39	NT
Beta3	22	NT
M1	3	NT
M2	11	NT
M3	15	NT
M4	21	NT
M5	13	NT
BZP rat brain site	5	NT
PBR	9	NT
SERT	32	NT
NET	80	634
DAT	–14	NT

a Receptors and transporters were initially tested with 10 μM **16f** and % inhibition measured compared to a known reference compound. Receptors and transporters with greater than 50% inhibition were selected for full assays to determine the affinity of **16f** for the receptor/transporter. Receptors with <100 nM affinity for **16f** in bold.

b NT – Not Tested due to <50% inhibition in primary assessment.

To determine its functional activity at 5-HT_1A_, 5-HT_2A_, and 5-HT_2B_, **16f** was tested in Gα_i_ or Gα_q_ calcium flux assays by Eurofins Discovery (Eurofins Cerep SA, Celle l’Evescault, France; Eurofins DiscoverX Corporation, Fremont, California). The results of these assays are listed in Table 5. When tested at 5-HT_1A_ in the Gα_i_ calcium flux assay, **16f** showed agonist activity with an estimated potency of 4.6 μM. The curve did not saturate at the highest tested concentration (10 μM), so the estimated *E*_max_ (58.8% of the serotonin response) may be an underestimate due to the low apparent potency of **16f** at 5-HT_1A_ for Gα_i_-coupled responses. Because agonist responses interfere with analysis of the antagonist mode of this assay, the IC_50_ of **16f** as >370 nM reflects the highest drug concentration not excluded from analysis. In Gα_q_ calcium flux assays, **16f** had no agonist activity at 5-HT_2A_ or 5-HT_2B_ (EC_50_ > 10 μM) and was a full antagonist at both receptors (5-HT_2A_*I*_max_ = 106%, IC_50_ = 532 nM; 5-HT_2B_*I*_max_ = 111%, IC_50_ = 770 nM). In comparison, **16f** had higher potency at D_4_R, with an IC_50_ of 69.3 nM in cAMP assays that is also responsive to Gα_i/o_-mediated signaling (Table 2). While one should be cautious about overinterpreting relative potencies collected across different assay conditions, these results suggest that **16f** is modestly D_4_R-selective over 5-HT_2A_ and 5-HT_2B_ with potential low potency agonism at 5-HT_1A_.

**Table 5:** Functional Assessment of **16f** and Control Compounds at 5-HT_1A_, 5-HT_2A_, and 5-HT_2B_ Receptors in Gα_i_ and Gα_q_ Calcium Flux Assays

**receptor**	**study mode**	**ligand**	**EC_50_/IC_50_ (nM)**	**max response** (% control)
5-HT_1A_	agonist	serotonin	3.40	100
	agonist	**16f**	4600	58.8
	antagonist	(S)-WAY-100635	6.60	100
	antagonist	**16f**	>370^a^	37.4
5-HT_2A_	agonist	serotonin	1.32	94.0
	agonist	**16f**	>10,000	0
	antagonist	altanserin HCl	5.27	101
	antagonist	**16f**	532	106
5-HT_2B_	agonist	serotonin	2.40	100
	agonist	**16f**	>10,000	14.1
	antagonist	LY 272015 HCl	0.37	100
	antagonist	**16f**	770	111

a 370 nM was the highest concentration of **16f** that was not excluded by Eurofins in the Gα_i_ assay as higher concentrations had agonist affects that interfere with interpretation of the antagonist assay.

### *In Silico* and *In Vitro* Pharmacokinetics Studies of **16f**

The potential for brain penetrance of **16f** was evaluated *in silico* by using central nervous system multiparameter optimization (CNS MPO) tools. **16f**, and the brain-penetrant CNS ligand buspirone as a comparator, had calculated CNS MPO scores of 4.5 and 5.8, respectively, shown in Supplemental Table S3; scores >4 correlate with CNS drug-like properties.^40^**16f** was also tested in Caco-2 membrane permeability assays (Eurofins Panlabs, St. Charles, Missouri) and the apical-to-basolateral (A-B) permeability of **16f** was 27 × 10^–6^ cm/s, comparable to the assay control compounds propranolol (24 × 10^–6^ cm/s) and buspirone (25 × 10^–6^ cm/s), correlated with high membrane permeability.

**Figure 3:**
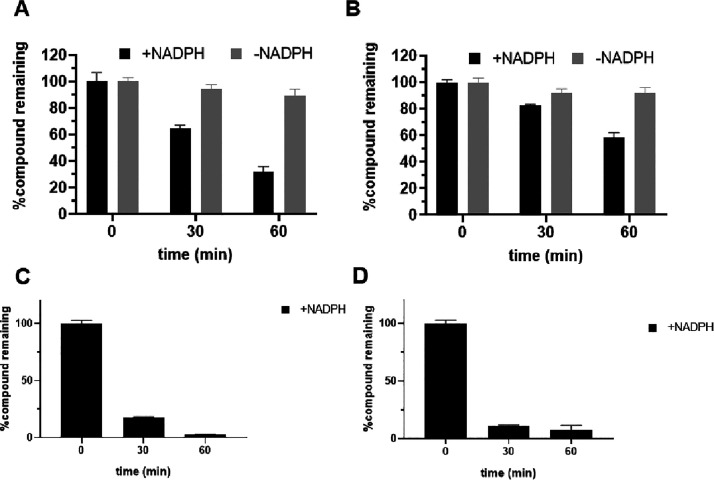
Phase I metabolic stability of **16f** in rat (A) and human (B) liver microsomes. **16f** shows time-dependent degradation in human and rat liver microsomes. **16f** was modestly stable in human liver microsomes. Data are expressed as mean ± SEM, *n* = 3. As a positive control for phase I metabolism, metabolic stability of buprenorphine in rat (C) and human (D) liver microsomes is also presented.

We then evaluated the phase I metabolic stability of **16f** using rat and human liver microsomes, as previously described.^41^ Incubation of **16f** with rat liver microsomes in the presence of NADPH resulted in time-dependent degradation, with ∼33% remaining after 1 h (Figure 3A). In human liver microsomes, **16f** showed greater stability, with ∼60% remaining after 1 h incubation (Figure 3B). These results predict that **16f** has modest liver metabolic stability in humans and relatively lower stability in the rat liver. HPLC traces of **16f** and the major metabolite of **16f**—a dealkylated 1-(pyridin-2-yl)piperazine product—are shown in Figure S2.

### Pharmacokinetic Assessment of **16f** in Rats

**Figure 4:**
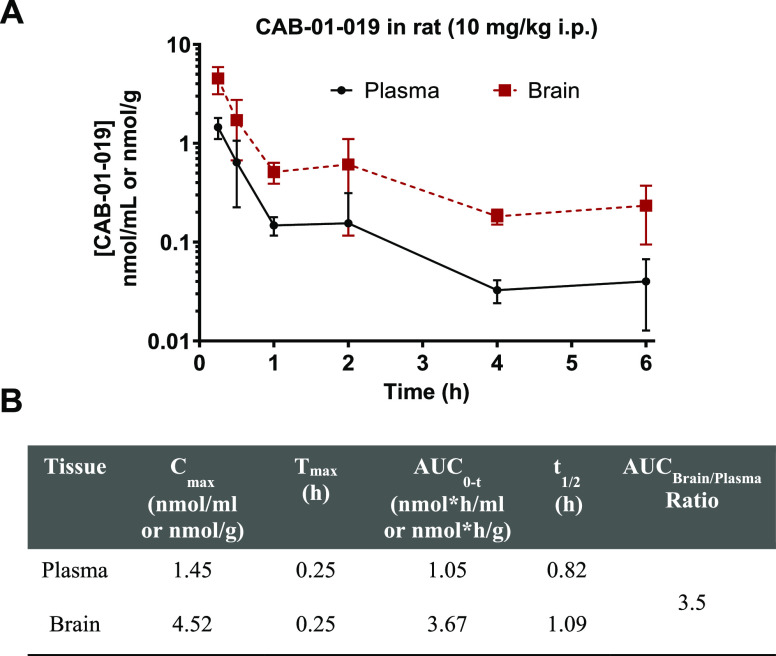
(A) Time-dependent *in vivo* pharmacokinetic analysis of **16f** (**CAB-01–019**) in Sprague–Dawley (SD) rats following intraperitoneal (ip) administration of 10 mg/kg **16f**. Data expressed as mean ± SEM, *n* = 3 for each time point. (B) Calculated pharmacokinetic parameters of **16f** in rats.

Given its adequate stability profile, we next evaluated the *in vivo* pharmacokinetic profile of **16f** in rats. Sprague–Dawley rats were dosed with **16f** (10 mg/kg, i.p.), and plasma and brain levels were measured 0–6 h postdose. The results from the pharmacokinetic analysis are listed in Figure 4A,B. **16f** demonstrated good exposure in both plasma and brain, with AUC_0–*t*_ values of 1.05 and 3.67 nmol·h/g, respectively. Compound **16f** was observed to have a brain penetration index (AUC_brain/plasma_ ratio) of 3.5 with an apparent half-life of ∼1 h (*t*_1/2_). The detailed pharmacokinetic parameters of **16f** are provided in Figure 4B.

### Behavioral Effects of **16f** in Rats Trained to Self-Administer Food and Cocaine

In order to test our hypothesis that D_4_R antagonism is a viable route for CUD pharmacotherapy, we evaluated whether **16f** altered cocaine self-administration, using food self-administration as a natural reward comparator. Separate groups of male Fischer 344 rats were trained to respond on a lever to receive food pellets or iv cocaine in multicomponent procedures. Both procedures included three components (60 min each for cocaine, 30 min each for food) in each test wherein the reinforcer was reduced across components (food: four, two, and one food pellets across components 1, 2, and 3, respectively; iv cocaine: 166, 83, and 41.5 mg/infusion across components 1, 2, and 3, respectively). After successful training, the saline vehicle and **16f** (5, 15, and 30 mg/kg, i.p.) were tested.

**Figure 5:**
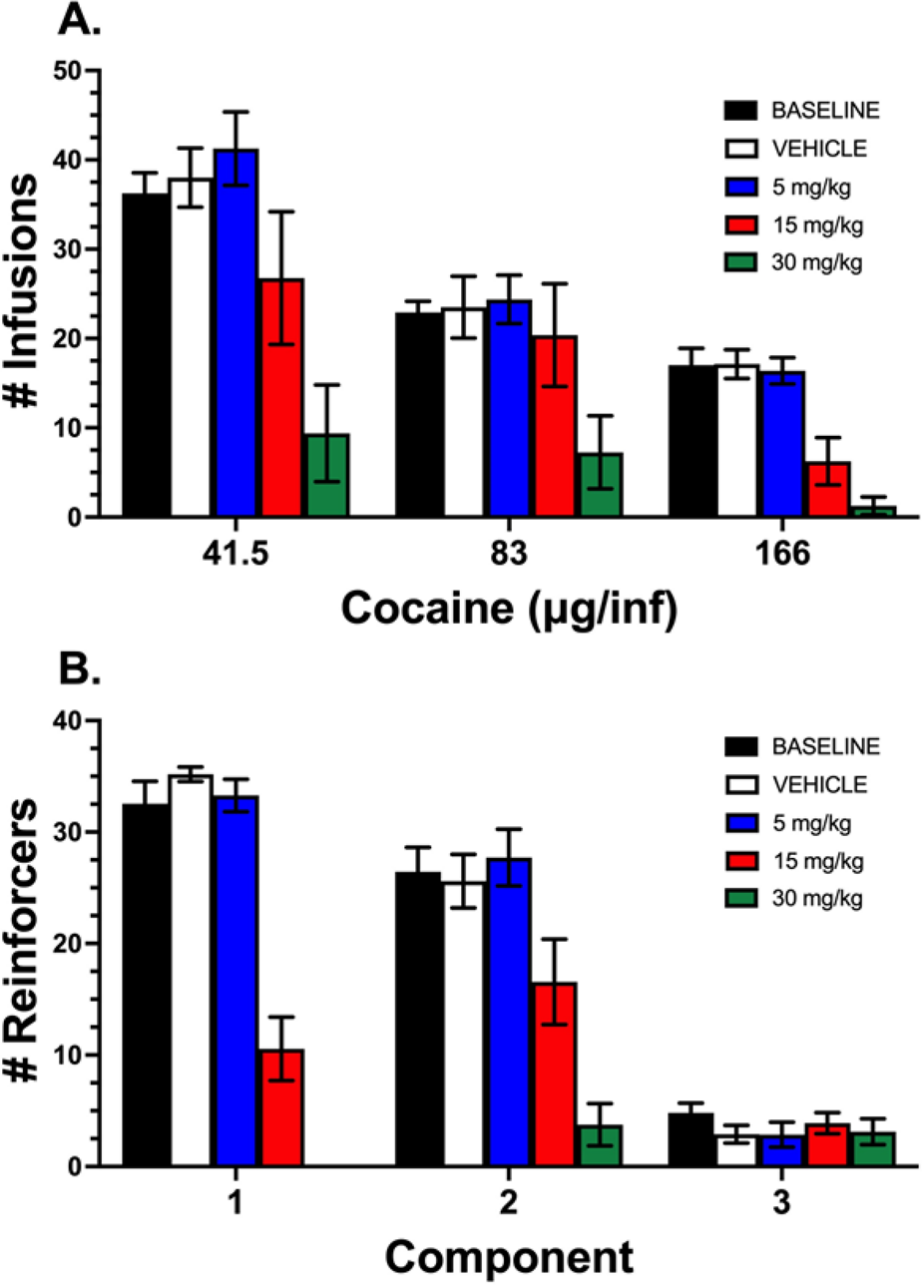
Effect of D_4_R antagonist **16f** on cocaine self-administration and food-maintained responding. (A). Number of infusions for each cocaine dose session at baseline, and following vehicle, 5, 15, and 30 mg/kg (i.p.) of **16f**. **16f** dose-dependently decreased intake at each cocaine dose in male Fisher F344 rats (*n* = 8 per group). (B). Number of food reinforcers for each component at baseline, and following vehicle, 5, 15, and 30 mg/kg (i.p.) of **16f**. **16f** dose-dependently decreased food-maintained responding in male Fisher F344 rats (*n* = 7–9 per group). Data expressed as mean ± SEM.

**16f** pretreatment produced a significant decrease in the number of infusions for each cocaine dose, an effect that was dependent upon the dose of the compound (5, 15, and 30 mg/kg, i.p.) (Figure 5A). Intake following saline pretreatment was not significantly different from baseline [*F*_2, 16_ = 0.2935, *P* = 0.75]. A significant main effect of compound **16f** on cocaine self-administration was observed [*F*_1.239, 34.69_ = 57.79, *P* < 0.0001] and a significant interaction of component and **16f** on cocaine intake [*F*_6, 56_ = 3.181, *P* = 0.0093]. The number of infusions obtained for each cocaine dose was significantly different after **16f** treatment, and the magnitude of effect was dependent on the dose of **16f** as well as the dose of cocaine self-administered.

Similarly, **16f** dose-dependently decreased food and maintained responding (Figure 5B). Intake following saline pretreatment was not significantly different from baseline [*F*_2, 32_ = 1.949, *P* = 0.1589]. A significant main effect of **16f** on food-maintained responding was observed [*F*_1.708, 49.53_ = 137.4, *P* < 0.0001] and a significant interaction of component and **16f** on cocaine intake [*F*_6, 58_ = 32.88, *P* < 0.0001].

Overall, these results indicate that **16f** is centrally active and reduces cocaine- and food-maintained responding. The effects of **16f** are most pronounced at lower unit doses of cocaine but at higher unit doses of food, suggesting some differentiation of these effects that will be more fully evaluated in follow-up studies. Future testing will also determine whether **16f** affects relapse-like responses and other behaviors relevant to CUD.

## Conclusions

Evidence from human genetic studies and animal models suggests D_4_R signaling modulates drug-taking and -seeking behaviors. Newer highly selective D_4_R antagonists will be useful to better characterize the role of D_4_R signaling *in vivo*, particularly in behavioral models of CUD. This study provided a detailed SAR analysis of a novel series of D_4_R partial agonists and antagonists. We identified several compounds with high D_4_R binding affinity and selectivity over other D_2_-like receptors (D_2_R, D_3_R) with diverse partial agonist and antagonist profiles. The low-efficacy D_4_R partial agonist **16f** was chosen as a lead compound suitable for pharmacokinetic and behavioral testing on the basis of its high selectivity over D_2_R and D_3_R. **16f** displayed acceptable *in vitro* metabolic stability in rat and human liver microsomes and good *in vivo* half-life and brain penetration parameters.

In behavioral testing, **16f** dose-dependently decreased cocaine- and food-maintained operant responses, with diverging effects on the reinforcer unit dose. These results suggest that D_4_R antagonism reduces the rewarding effects of cocaine and is a plausible route for CUD pharmacotherapy development. We cannot rule out the importance of off-target effects in the behavioral response to **16f**—the compound is only modestly D_4_R-selective over its antagonistic effects at 5-HT_2A_ and 5-HT_2B_ receptors. This may actually represent a favorable profile, as prior studies have reported that 5-HT_2A_ and 5-HT_2B_ antagonists can attenuate cocaine-taking and cocaine-seeking behaviors.^42−46^ Our results also suggest the possibility of **16f** producing low-potency agonism at 5-HT_1A_, which has been previously reported to increase the reinforcing strength of a low cocaine dose.^47^ This is at odds with our behavioral results, which seem to indicate a stronger effect of **16f** at lower unit doses of cocaine.

The extended analogue library created while analyzing **16f***in vitro* and *in vivo* identified several additional modifications that improved D_4_R affinity and selectivity over D_2_R and D_3_R; future analyses will determine whether these modifications alter activity at 5-HT_2A_, 5-HT_2B_, and 5-HT_1A_. We are optimistic that these analogues will be useful as novel *in vivo* research tools, and we plan to examine additional ADME characteristics of selected library members. It is interesting to speculate that a collection of ligands with varying efficacies may allow for the fine-tuning of D_4_R inhibition, potentially leading to a fuller understanding of functional consequences of D_4_R signaling levels in the development of therapeutics for SUDs and other neuropsychiatric disorders.

## Experimental Methods

Reaction conditions and yields were not optimized. Anhydrous solvents were purchased from Aldrich and used without further purification. All other chemicals and reagents were purchased from Sigma-Aldrich Co., LLC, Aurora Fine Chemicals LLC, VWR Chemicals, Enamine, Acros Organics, and Alfa Aesar. All amine final products were converted to either the oxalate or hydrochloride salt. Spectroscopic data and yields refer to the free base forms of compounds. Flash chromatography was performed using silica gel (EMD Chemicals, Inc.; 230–400 mesh, 60 Å) by using a Teledyne Isco CombiFlash RF system. ^1^H and ^13^C spectra were acquired by using a JEOL ECZ-400S NMR spectrometer. ^1^H chemical shifts are reported as parts per million (δ ppm) relative to tetramethylsilane (0.00 ppm). All of the coupling constants are measured in Hz. Chemical shifts for ^13^C NMR spectra are reported as parts per million (δ ppm) and referenced according to deuterated solvent for ^1^H spectra (CDCl_3_, 7.26, or CD_3_OD, 3.31) and ^13^C spectra (CDCl_3_, 77.1, or CD_3_OD, 49.0). Chemical shifts, multiplicities, and coupling constants (J) were reported and calculated using Mnova 64. Combustion analysis was performed by Atlantic Microlab, Inc. (Norcross, Georgia), and the results agree within ±0.4% of calculated values (Table S1). cLogP and PSA values were calculated using ChemDraw version 20.0. Melting point determination was conducted using an SRS OptiMelt MPA100-Automated melting point apparatus and are uncorrected. Based on NMR and combustion analysis data, all final compounds are ≥95% pure. All compounds within this series are covered under an existing patent,^32^ but only **16a** and **16e**^31^ have been previously described in the peer-reviewed literature.

### General Method A^31^

4-Chlorobutanoyl chloride or 5-chloropentanoyl chloride (1.24 equiv) was added dropwise to a solution of substituted or unsubstituted 2-aminobenzenethiol (1.00 equiv) in toluene at 0 °C over 15 min, and an off-white precipitate was formed. The reaction mixture was stirred at room temperature for 48 h, under a N_2_ atmosphere. After the reaction was complete, the solvent was removed *in vacuo.* The crude mixture was diluted with aqueous NaHCO_3_ (100 mL) and EtOAc (100 mL), and the two layers were separated and then extracted with EtOAc (2 × 100 mL) and washed with brine (100 mL). The combined organic layer was dried over Na_2_SO_4_, filtered, and concentrated. The product was purified by flash column chromatography (5–95% EtOAc:hexanes) gradient to give the desired substituted or unsubstituted 2-(3-chloropropyl)benzo[*d*]thiazole or 2-(4-chlorobutyl)benzo[*d*]thiazole compounds.

#### 2-(4-Chlorobutyl)benzo[*d*]thiazole (**15a**)^31^

Compound **15a** was synthesized as described for general method A by using 5-chloropentanoyl chloride (5.87 g, 49.5 mmol) and 2-aminobenzenethiol (4.27 mL, 39.9 mmol) in toluene (150 mL). Product **15a** is formed as a brown sticky oil (5.98 g, 66% yield). ^1^H NMR (CD_3_OD) δ 8.26 (d, *J* = 8.1 Hz, 1H), 8.16 (d, *J* = 8.5 Hz, 1H), 7.94–7.84 (m, 1H), 7.80 (t, *J* = 7.7 Hz, 1H), 4.83–4.81 (m, 2H), 4.62 (t, *J* = 6.1 Hz, 2H), 2.38–2.27 (m, 2H), 2.20–2.10 (m, 2H).

#### 2-(3-Chloropropyl)benzo[*d*]thiazole (**15b**)^31^

Compound **15b** was synthesized as described for general method **A** by using 4-chlorobutanoyl chloride (5.54 mL, 49.53 mmol) and 2-aminobenzenethiol (4.27 mL, 39.9 mmol) in toluene (150 mL). Product **15b** formed as a greenish oil (6.30 g, 75% yield). ^1^H NMR (CDCl_3_) δ 7.96 (dd, *J* = 8.0, 1.5 Hz, 1H), 7.83 (dt, *J* = 8.5, 1.2 Hz, 1H), 7.44 (ddq, *J* = 8.2, 7.1, 1.1 Hz, 1H), 7.34 (ddt, *J* = 8.2, 7.1, 1.0 Hz, 1H), 3.66 (td, *J* = 6.2, 1.0 Hz, 2H), 3.27 (td, *J* = 7.3, 1.2 Hz, 2H), 2.41–2.30 (m, 2H).

#### 2-(3-Chloropropyl)-6-methylbenzo[*d*]thiazole (**18a**)

Compound **18a** was synthesized as described for general method A by using 4-chlorobutanoyl chloride (1.00 mL, 8.91 mmol) and 2-amino-5-methylbenzenethiol (1.00 g, 7.18 mmol) in toluene (50 mL). Product **18a** formed as a yellowish oil (1.39 g, 86% yield). ^1^H NMR (CDCl_3_) δ 7.86 (s, 1H), 7.83 (d, *J* = 8.2 Hz, 1H), 7.60 (d, *J* = 14.5 Hz, 1H), 3.66 (td, *J* = 6.0, 1.7 Hz, 2H), 3.31–3.03 (m, 2H), 2.51 (d, *J* = 38.0 Hz, 3H), 2.40–2.22 (m, 2H).

#### 2-(3-Chloropropyl)-7-methoxybenzo[*d*]thiazole (**18b**)

Compound **18b** was synthesized as described for general method A by using 4-chlorobutanoyl chloride (0.89 mL, 7.98 mmol) and 2-amino-6-methoxybenzenethiol (1.00 g, 6.44 mmol) in toluene (100 mL). Product **18b** formed as a black solid (1.05 g, 67% yield). ^1^H NMR (CDCl_3_) δ 7.59 (dt, *J* = 8.1, 0.8 Hz, 1H), 7.39 (td, *J* = 8.0, 0.7 Hz, 1H), 6.80 (d, *J* = 8.0 Hz, 1H), 3.97 (s, 3H), 3.66 (t, *J* = 6.4 Hz, 2H), 3.28 (t, *J* = 7.3 Hz, 2H), 2.43–2.30 (m, 2H).

#### 2-(3-Chloropropyl)-6-methoxybenzo[*d*]thiazole (**18c**)

Compound **18c** was synthesized as described for general method A by using 4-chlorobutanoyl chloride (0.89 mL, 7.99 mmol) and 2-amino-5-methoxybenzenethiol (1.00 g, 6.44 mmol) in toluene (50 mL). Product **18c** is formed as a dark-brown solid (890 mg, 57% yield). ^1^H NMR (CD_3_OD) δ 7.94 (d, *J* = 9.1 Hz, 1H), 7.87–7.69 (m, 1H), 7.18–6.98 (m, 1H), 4.83 (s, 3H), 3.76–3.59 (m, 2H), 3.33–3.18 (m, 2H), 2.91 (p, *J* = 7.7 Hz, 2H).

#### 6-Chloro-2-(3-chloropropyl)benzo[*d*]thiazole (**18d**)

Compound **18d** was synthesized as described for general method A by using 4-chlorobutanoyl chloride (0.87 mL, 7.77 mmol) and 2-amino-5-chlorobenzenethiol (1.00 g, 6.26 mmol) in toluene (50 mL). Product **18d** is formed as a brown solid (1.13 g, 73% yield). ^1^H NMR (CDCl_3_) δ 7.89–7.80 (m, 2H), 7.43–7.40 (m, 1H), 3.67 (t, *J* = 6.3 Hz, 2H), 3.27 (t, *J* = 7.4 Hz, 2H), 2.42–2.31 (m, 2H).

#### 5-Chloro-2-(3-chloropropyl)benzo[*d*]thiazole (**18e**)

Compound **18e** was synthesized as described for general method A by using 4-chlorobutanoyl chloride (1.74 mL, 15.5 mmol) and 2-amino-4-chlorobenzenethiol (2.00 g, 12.5 mmol) in toluene (75 mL). Product **18e** is formed as a yellowish solid (1.73 g, 56% yield). ^1^H NMR (CDCl_3_) δ: 7.95 (d, *J* = 2.0 Hz, 1H), 7.74 (d, *J* = 8.5 Hz, 1H), 7.34 (dd, *J* = 8.5, 2.0 Hz, 1H), 3.67 (t, *J* = 6.3 Hz, 2H), 3.28 (t, *J* = 7.4 Hz, 2H), 2.36 (tt, *J* = 7.5, 6.3 Hz, 2H).

#### 4-Chloro-2-(3-chloropropyl)benzo[*d*]thiazole (**18f**)

Compound **18f** was synthesized as described for general method A by using 4-chlorobutanoyl chloride (1.74 mL, 15.5 mmol) and 2-amino-3-chlorobenzenethiol (2.00 g, 12.5 mmol) in toluene (100 mL). Product **18f** is formed as a black solid (1.85 g, 60% yield). ^1^H NMR (CDCl_3_) δ 7.89–7.78 (m, 1H), 7.44–7.38 (m, 1H), 7.25 (dd, *J* = 2.9, 1.8 Hz, 1H), 3.67 (t, *J* = 6.3 Hz, 2H), 3.33–3.21 (m, 2H), 2.43–2.29 (m, 2H).

### General Method B

Substituted or unsubstituted 2-(3-chloropropyl)benzo[*d*]thiazole or 2-(4-chlorobutyl)benzo[*d*]thiazole (1.0 equiv) was added to a solution of K_2_CO_3_ (10 equiv), KI (0.1 equiv), and substituted or unsubstituted arylpiperidinyl or arylpiperazinyl (1.2 equiv) in an anhydrous acetonitrile solution. The reaction mixture was stirred at reflux (80 °C) for 20 h, under a N_2_ atmosphere. The reaction mixture was cooled to room temperature, and the solvent was removed *in vacuo*. The residue was diluted with water (100 mL) and dichloromethane (DCM) (100 mL) and then extracted with DCM (3 × 100 mL) and washed with brine (100 mL). The combined organic layer was dried over Na_2_SO_4_, filtered, and then evaporated to afford the crude products. All final products were purified by flash column chromatography eluting with 5% CMA (95% chloroform, 4% methanol, 1% ammonium hydroxide) gradient to give the desired compounds.

#### 2-(4-(4-(Pyrimidin-2-yl)piperazin-1-yl)butyl)benzo[*d*]thiazole (**16a**)

Compound **16a** was synthesized as described for general method B by using K_2_CO_3_ (4.28 g, 31.0 mmol), KI (52 mg), 2-(4-chlorobutyl)benzo[*d*]thiazole (**15a**) (700 mg, 3.10 mmol), and 2-(piperazin-1-yl)pyrimidine (0.53 mL, 3.72 mmol) in an anhydrous acetonitrile (18 mL) solution. The crude product was purified by flash column chromatography to obtain pure **16a** as a cream solid (320 mg, 29% yield). ^1^H NMR (400 MHz CDCl_3_) δ 8.26 (d, *J* = 4.8 Hz, 2H), 7.93 (d, *J* = 8.0 Hz, 1H), 7.80 (d, *J* = 7.6 Hz, 1H), 7.43–7.39 (m, 1H), 7.33–7.29 (m, 1H), 6.44–6.41 (m, 1H), 3.80–3.78 (m, 4H), 3.13 (t, *J* = 7.6 Hz, 2H), 2.46–2.38 (m, 6H), 1.92 (p, *J* = 7.2 Hz, 2H), 1.65 (p, *J* = 7.6 Hz, 2H). ^13^C NMR (101 MHz, CDCl_3_) δ: 171.85, 161.61, 157.66, 153.20, 135.09, 125.88, 124.67, 122.50, 121.47, 109.76, 58.19, 53.09, 43.63, 34.12, 27.55, 26.27. The HCl salt was precipitated from 2-propanol. Mp 239–241 °C. Anal. (C_19_H_23_N_5_S•2HCl•0.5H_2_O) C, H, N.

#### 2-(4-(4-(Pyridin-2-yl)piperazin-1-yl)butyl)benzo[*d*]thiazole (**16b**)

Compound **16b** was synthesized as described for general method B by using K_2_CO_3_ (4.28 g, 31.0 mmol), KI (52 mg), 2-(4-chlorobutyl)benzo[*d*]thiazole (**15a**) (700 mg, 3.10 mmol), and 1-(pyridin-2-yl)piperazine (0.57 mL, 3.72 mmol) in an anhydrous acetonitrile (18 mL) solution. The crude product was purified by flash column chromatography to obtain pure **16b** as a cream solid (313 mg, 29% yield). ^1^H NMR (400 MHz CDCl_3_) δ 8.18–8.17 (m, 1H), 7.97–7.94 (m, 1H), 7.84–7.82 (m, 1H), 7.47–7.42 (m, 2H), 7.36–7.34 (m, 1H), 6.63–6.58 (m, 2H), 3.54–3.51 (m, 4H), 3.16 (t, *J* = 7.6 Hz, 2H), 2.54 (t, *J* = 4.8 Hz, 4H), 2.45–2.42 (m, 2H), 1.92 (p, *J* = 8.0 Hz, 2H), 1.68 (p, *J* = 7.6 Hz, 2H). ^13^C NMR (101 MHz, CDCl_3_) δ: 171.90, 159.54, 153.22, 147.94, 137.41, 135.11, 125.91, 124.69, 122.52, 121.50, 113.24, 107.02, 58.21, 53.08, 45.18, 34.15, 27.60, 26.31. The HCl salt was precipitated from 2-propanol. Mp 235–237 °C. Anal. (C_20_H_24_N_4_S•3HCl•1.5H_2_O) C, H, N.

#### 2-(4-(4-(5-Methylpyridin-2-yl)piperazin-1-yl)butyl)benzo[*d*]thiazole (**16c**)

Compound **16c** was synthesized as described for general method B by using K_2_CO_3_ (1.96 g, 14.2 mmol), KI (24 mg), 2-(4-chlorobutyl)benzo[*d*]thiazole (**15a**) (320 mg, 1.42 mmol), and 1-(5-methylpyridin-2-yl)piperazine (302 mg, 1.70 mmol) in an anhydrous acetonitrile (8 mL) solution. The crude product was purified by flash column chromatography to obtain pure **16c** as a light-brown solid (153 mg, 31% yield). ^1^H NMR (400 MHz CDCl_3_) δ 8.01 (s, 1H), 7.96 (d, *J* = 7.2 Hz, 1H), 7.85–7.983 (m, 1H), 7.45–7.43 (m, 1H), 7.36–7.29 (m, 2H), 6.57 (d, *J* = 8.4 Hz, 1H), 3.48 (t, *J* = 5.2 Hz, 4H), 3.17 (t, *J* = 7.6 Hz, 2H), 2.55 (t, *J* = 5.2 Hz, 4H), 2.46–2.42 (m, 2H), 2.19 (s, 3H), 1.94 (p, *J* = 7.6 Hz, 2H), 1.65 (p, *J* = 8.0 Hz, 2H). ^13^C NMR (101 MHz, CDCl_3_) δ: 171.92, 158.14, 153.22, 147.66, 138.35, 135.12, 125.90, 124.68, 122.52, 122.29, 121.50, 106.97, 58.24, 53.09, 45.69, 34.16, 27.62, 26.33, 17.33. The HCl salt was precipitated from 2-propanol. Mp 200–202 °C. Anal. (C_21_H_26_N_4_S•3HCl•1.25H_2_O) C, H, N.

#### 2-(4-(4-(5-Chloropyridin-2-yl)piperazin-1-yl)butyl)benzo[*d*]thiazole (**16d**)

Compound **16d** was synthesized as described for general method B by using K_2_CO_3_ (3.06 g, 22.2 mmol), KI (37 mg), 2-(4-chlorobutyl)benzo[*d*]thiazole (**15a**) (500 mg, 2.22 mmol), and 1-(5-chloropyridin-2-yl)piperazine (525 mg, 2.66 mmol) in an anhydrous acetonitrile (13 mL) solution. The crude product was purified by flash column chromatography to obtain pure **16d** as a white solid (202 mg, 24% yield). ^1^H NMR (400 MHz CDCl_3_) δ 8.09 (q, *J* = 2.4 Hz, 1H), 7.99–7.91 (m, 1H), 7.86–7.79 (m, 1H), 7.49–7.31 (m, 3H), 6.56 (dt, *J* = 9.2, 2.3 Hz, 1H), 3.50 (q, *J* = 4.4 Hz, 4H), 3.19–3.11 (m, 2H), 2.56–2.49 (m, 4H), 22.39 (m, 2H), 1.92 (q, *J* = 7.9 Hz, 2H), 1.72–1.60 (m, 2H). ^13^C NMR (101 MHz, CDCl_3_) δ: 171.96, 157.91, 153.33, 146.33, 137.18, 135.21, 126.03, 124.81, 122.62, 121.60, 120.22, 107.83, 58.22, 52.98, 45.37, 34.22, 31.03, 27.64. The Oxalate salt was precipitated from 2-propanol/acetone. Mp 214–215 °C. Anal. (C_20_H_23_ClN_4_S•C_2_H_2_O_4_) C, H, N.

#### 2-(3-(4-(Pyrimidin-2-yl)piperazin-1-yl)propyl)benzo[*d*]thiazole (**16e**)

Compound **16e** was synthesized as described for general method B by using K_2_CO_3_ (4.66 g, 33.7 mmol), KI (60 mg), 2-(3-chloropropyl)benzo[*d*]thiazole (**15b**) (714 mg, 3.37 mmol), and 2-(piperazin-1-yl)pyrimidine (0.57 mL, 4.04 mmol) in an anhydrous acetonitrile (20 mL) solution. The crude product was purified by flash column chromatography to obtain pure **16e** as a brown oil (480 mg, 42% yield). ^1^H NMR (400 MHz CDCl_3_) δ 8.27 (d, *J* = 4.8 Hz, 2H), 7.96 (d, *J* = 8.4 Hz, 1H), 7.82–7.80 (m, 1H), 7.44–7.39 (m, 1H), 7.34–7.29 (m, 1H), 6.45–6.42 (t, *J* = 4.8 Hz, 1H), 3.81–3.79 (m, 4H), 3.17 (t, *J* = 7.6 Hz, 2H), 2.49–2.46 (m, 6H), 2.11 (p, *J* = 7.2 Hz, 2H). ^13^C NMR (101 MHz, CDCl_3_) δ: 171.68, 161.60, 157.67, 153.21, 135.13, 125.89, 124.69, 122.50, 121.48, 109.77, 57.44, 53.01, 43.64, 32.08, 26.68. The HCl salt was precipitated from 2-propanol. Mp 182–184 °C. Anal. (C_18_H_21_N_5_S•2HCl•1.75H_2_O) C, H, N.

#### 2-(3-(4-(Pyridin-2-yl)piperazin-1-yl)propyl)benzo[*d*]thiazole (**16f**)

Compound **16f** was synthesized as described for general method B by using K_2_CO_3_ (4.56 g, 33.0 mmol), KI (55 mg), 2-(3-chloropropyl)benzo[*d*]thiazole (**15b**) (700 mg, 3.30 mmol), and 1-(pyridin-2-yl)piperazine (0.53 mL, 3.72 mmol) in an anhydrous acetonitrile (20 mL) solution. The crude product was purified by flash column chromatography to obtain pure **16f** as a brown oil (530 mg, 47% yield). ^1^H NMR (400 MHz CDCl_3_) δ 8.18–8.16 (m, 1H), 7.96 (d, *J* = 7.2 Hz, 1H), 7.83–7.81 (m, 1H), 7.46–7.41 (m, 2H), 7.35–7.31 (m, 1H), 6.62–6.57 (m, 2H), 3.54–3.51 (m, 4H), 3.18 (t, *J* = 7.2 Hz, 2H), 2.56–2.48 (m, 6H), 2.11 (p, *J* = 7.6 Hz, 2H). ^13^C NMR (101 MHz, CDCl_3_) δ: 171.70, 159.52, 153.23, 147.94, 137.42, 135.16, 125.91, 124.70, 122.51, 121.49, 113.24, 107.02, 57.43, 52.97, 45.19, 32.09, 26.73. The HCl salt was precipitated from 2-propanol. Mp 245–247 °C. Anal. (C_19_H_22_N_4_S•3HCl•2H_2_O) C, H, N.

#### 2-(3-(4-(4-Methylpyridin-2-yl)piperazin-1-yl)propyl)benzo[*d*]thiazole (**19a**)

Compound **19a** was synthesized as described for general method B by using K_2_CO_3_ (4.89 g, 35.4 mmol), KI (59 mg), 2-(3-chloropropyl)benzo[*d*]thiazole (**15b**) (750 mg, 3.54 mmol), and 1-(4-methylpyridin-2-yl)piperazine (754 mg, 4.25 mmol) in an anhydrous acetonitrile (21 mL) solution. The crude product was purified by flash column chromatography to obtain pure **19a** as a brown oil (360 mg, 29% yield). ^1^H NMR (400 MHz CDCl_3_) δ 8.04 (d, *J* = 4.9 Hz, 1H), 7.96 (d, *J* = 8.1 Hz, 1H), 7.86–7.81 (m, 1H), 7.46–7.82 (m, 1H), 7.34 (td, *J* = 7.6, 1.2 Hz, 1H), 6.46 (d, *J* = 5.9 Hz, 2H), 3.53 (t, *J* = 5.1 Hz, 4H), 3.18 (t, *J* = 7.6 Hz, 2H), 2.61–2.48 (m, 6H), 2.25 (s, 3H), 2.13 (p, *J* = 7.5 Hz, 2H).). ^13^C NMR (101 MHz, CDCl_3_) δ 171.73, 159.89, 153.33, 148.51, 147.67, 135.27, 126.02, 124.82, 122.63, 121.60, 115.03, 107.65, 57.53, 53.06, 45.46, 32.18, 26.69, 21.53. The HCl salt was precipitated from 2-propanol. Mp 213-215 °C. Anal. (C_20_H_24_N_4_S•3HCl•1.75H_2_O) C, H, N.

#### 2-(3-(4-(3-Methoxypyridin-2-yl)piperazin-1-yl)propyl)benzo[*d*]thiazole (**19b**)

Compound **19b** was synthesized as described for general method B by using K_2_CO_3_ (4.89 g, 35.4 mmol), KI (41 mg), 2-(3-chloropropyl)benzo[*d*]thiazole (**15b**) (523 mg, 2.47 mmol), and 1-(3-methoxypyridin-2-yl)piperazine (570 mg, 2.97 mmol) in an anhydrous acetonitrile (15 mL) solution. The crude product was purified by flash column chromatography to obtain pure **19b** as a brown oil (380 mg, 42% yield). ^1^H NMR (400 MHz CDCl_3_) δ 7.98–7.92 (m, 1H), 7.88–7.80 (m, 2H), 7.46–7.42 (m, 1H), 7.36–7.32 (m, 1H), 7.01 (dd, *J* = 8.0, 1.5 Hz, 1H), 6.82 (dd, *J* = 7.9, 4.9 Hz, 1H), 3.83 (s, 3H), 3.45 (s, 4H), 3.18 (t, *J* = 7.5 Hz, 2H), 2.62 (d, *J* = 40.6 Hz, 6H), 2.08–2.05 (m, 2H). ^13^C NMR (101 MHz, CDCl_3_) δ: 146.87, 138.96, 135.28, 126.04, 124.88, 122.64, 121.63, 117.57, 74.55, 55.36, 34.21, 31.83, 29.62, 29.30, 25.41, 25.36, 22.67, 14.16. The HCl salt was precipitated from 2-propanol. Mp 154–156 °C. Anal. (C_20_H_24_N_4_OS•3HCl•1.5H_2_O) C, H, N.

#### 6-Methyl-2-(3-(4-(pyridin-2-yl)piperazin-1-yl)propyl)benzo[*d*]thiazole (**19c**)

Compound **19c** was synthesized as described for general method B by using K_2_CO_3_ (4.28 g, 31.1 mmol), KI (52 mg), 2-(3-chloropropyl)-6-methylbenzo[*d*]thiazole (**18a**) (700 mg, 3.10 mmol), and 1-(pyridin-2-yl)piperazine (607 mg, 3.72 mmol) in an anhydrous acetonitrile (18 mL) solution. The crude product was purified by flash column chromatography to obtain pure **19c** as a light-brown solid (460 mg, 42% yield). ^1^H NMR (400 MHz CDCl_3_) δ 8.17 (ddd, *J* = 4.9, 2.0, 0.9 Hz, 1H), 7.82 (d, *J* = 8.3 Hz, 1H), 7.61 (s, 1H), 7.45 (ddd, *J* = 8.9, 7.1, 1.9 Hz, 1H), 7.23 (d, *J* = 1.7 Hz, 1H), 6.67–6.56 (m, 2H), 3.53 (t, *J* = 5.1 Hz, 4H), 3.14 (t, *J* = 7.6 Hz, 2H), 2.56 (t, *J* = 5.1 Hz, 4H), 2.50 (dd, *J* = 8.4, 6.3 Hz, 2H), 2.45 (s, 3H), 2.10 (p, *J* = 7.5 Hz, 2H). ^13^C NMR (101 MHz, CDCl_3_) δ: 170.67, 159.62, 151.40, 148.04, 137.56, 135.40, 134.86, 127.55, 122.08, 121.38, 113.38, 107.16, 57.56, 53.06, 45.26, 32.13, 26.79, 21.56. The HCl salt was precipitated from 2-propanol. Mp 230–231 °C. Anal. (C_20_H_24_N_4_S•3HCl•2H_2_O) C, H, N.

#### 6-Chloro-2-(3-(4-(pyridin-2-yl)piperazin-1-yl)propyl)benzo[*d*]thiazole (**19d**)

Compound **19d** was synthesized as described for general method B by using K_2_CO_3_ (5.05 g, 36.6 mmol), KI (61 mg), 6-chloro-2-(3-chloropropyl)benzo[*d*]thiazole (**18d)**(900 mg, 3.66 mmol), and 1-(pyridin-2-yl)piperazine (716 mg, 4.39 mmol) in an anhydrous acetonitrile (22 mL) solution. The crude product was purified by flash column chromatography to obtain pure **19d** as a brown solid (510 mg, 38% yield). ^1^H NMR (400 MHz CDCl_3_) δ 8.17 (dd, *J* = 4.9, 2.1 Hz, 1H), 7.88–7.78 (m, 2H), 7.49–7.38 (m, 2H), 6.65–6.58 (m, 2H), 3.53 (t, *J* = 5.1 Hz, 4H), 3.17 (t, *J* = 7.5 Hz, 2H), 2.62–2.47 (m, 6H), 2.15–2.05 (m, 2H). ^13^C NMR (101 MHz, CDCl_3_) δ: 172.36, 159.61, 151.90, 148.06, 137.56, 136.47, 130.73, 126.80, 123.35, 121.23, 113.41, 107.16, 57.43, 53.05, 45.27, 32.15, 26.62. The HCl salt was precipitated from 2-propanol. Mp 233–235 °C. Anal. (C_19_H_21_ClN_4_S_3_•3HCl•0.5H_2_O) C, H, N.

#### 5-Chloro-2-(3-(4-(pyridin-2-yl)piperazin-1-yl)propyl)benzo[*d*]thiazole (**19e**)

Compound **19e** was synthesized as described for general method **B** by using K_2_CO_3_ (5.61 g, 40.6 mmol), KI (67 mg), 5-chloro-2-(3-chloropropyl)benzo[*d*]thiazole (**18e**) (1.00 g, 4.06 mmol), and 1-(pyridin-2-yl)piperazine (796 mg, 4.88 mmol) in an anhydrous acetonitrile (24 mL) solution. The crude product was purified by flash column chromatography to obtain pure **19e** as a light-brown solid (650 mg, 43% yield). ^1^H NMR (400 MHz CDCl_3_) δ 8.21–8.13 (m, 1H), 7.93 (d, *J* = 2.1 Hz, 1H), 7.73 (d, *J* = 8.5 Hz, 1H), 7.49–7.41 (m, 1H), 7.31 (dd, *J* = 8.6, 2.1 Hz, 1H), 6.66–6.57 (m, 2H), 3.53 (t, *J* = 5.0 Hz, 4H), 3.17 (t, *J* = 7.5 Hz, 2H), 2.62–2.45 (m, 6H), 2.11 (p, *J* = 7.4 Hz, 2H). ^13^C NMR (101 MHz, CDCl_3_) δ: 173.84, 159.61, 154.23, 148.05, 137.55, 133.53, 132.05, 125.31, 122.54, 122.28, 113.41, 107.16, 57.43, 53.05, 45.27, 32.24, 26.65. The HCl salt was precipitated from 2-propanol. Mp 239–241 °C. Anal. (C_19_H_21_ClN_4_S•3HCl•1.75H_2_O) C, H, N.

#### 4-Chloro-2-(3-(4-(pyridin-2-yl)piperazin-1-yl)propyl)benzo[*d*]thiazole (**19f**)

Compound **19f** was synthesized as described for general method B by using K_2_CO_3_ (5.61 g, 40.6 mmol), KI (67 mg), 4-chloro-2-(3-chloropropyl)benzo[*d*]thiazole (**18f**) (1.00 g, 4.06 mmol), and 1-(pyridin-2-yl)piperazine (796 mg, 4.88 mmol) in an anhydrous acetonitrile (24 mL) solution. The crude product was purified by flash column chromatography to obtain pure **19f** as a cream solid (830 mg, 55% yield). ^1^H NMR (400 MHz CDCl_3_) δ 8.17 (ddd, *J* = 4.9, 2.1, 1.0 Hz, 1H), 7.88–7.78 (m, 2H), 7.49–7.37 (m, 2H), 6.62 (ddt, *J* = 8.4, 7.2, 2.9 Hz, 2H), 3.54 (t, *J* = 5.0 Hz, 4H), 3.17 (t, *J* = 7.5 Hz, 2H), 2.55 (dt, *J* = 24.6, 6.2 Hz, 6H), 2.12 (p, *J* = 7.3 Hz, 2H). ^13^C NMR (101 MHz, CDCl_3_) δ: 172.15, 159.48, 151.89, 148.06, 137.61, 136.47, 130.76, 126.82, 123.36, 121.24, 113.52, 107.19, 57.38, 52.95, 45.10, 32.08, 26.36. The HCl salt was precipitated from 2-propanol. Mp 233–234 °C. Anal. (C_19_H_21_ClN_4_S•3HCl•0.25H_2_O) C, H, N.

#### 2-(3-(4-(3-Methylpyridin-2-yl)piperidin-1-yl)propyl)benzo[*d*]thiazole (**20a**)

Compound **20a** was synthesized as described for general method B by using K_2_CO_3_ (3.66 g, 26.5 mmol), KI (44 mg), 2-(3-chloropropyl)benzo[*d*]thiazole (**15b**) (561 mg, 2.65 mmol), and 3-methyl-2-(piperidin-4-yl)pyridine (560 mg, 3.18 mmol) in an anhydrous acetonitrile (16 mL) solution. The crude product was purified by flash column chromatography to obtain pure **20a** as a brown oil (680 mg, 73% yield). ^1^H NMR (400 MHz CDCl_3_) δ 8.38 (dd, *J* = 4.8, 1.7 Hz, 1H), 7.95 (dt, *J* = 8.1, 0.9 Hz, 1H), 7.85–7.79 (m, 1H), 7.42 (ddd, *J* = 8.2, 7.2, 1.3 Hz, 1H), 7.38–7.29 (m, 2H), 6.98 (dd, *J* = 7.6, 4.7 Hz, 1H), 3.17 (t, *J* = 7.6 Hz, 2H), 3.06 (dd, *J* = 10.6, 3.1 Hz, 2H), 2.82 (tt, *J* = 10.9, 3.6 Hz, 1H), 2.48 (t, *J* = 7.2 Hz, 2H), 2.30 (s, 3H), 2.12–2.03 (m, 4H), 1.75–1.67 (m, 2H), 1.31–1.22 (m, 2H). ^13^C NMR (101 MHz, CDCl_3_) δ: 172.26, 162.81, 153.35, 146.91, 137.75, 135.35, 130.28, 125.92, 124.70, 122.56, 121.60, 121.06, 57.55, 54.25, 40.52, 32.10, 30.91, 27.07, 18.73. The oxalate salt was precipitated from 2-propanol. Mp 167–168 °C. Anal. (C_21_H_25_N_3_S• C_2_H_2_O_4_•0.25H_2_O•0.75C_3_H_7_OH) C, H, N.

#### 2-(3-(4-(5-Methoxypyridin-2-yl)piperidin-1-yl)propyl)benzo[*d*]thiazole (**20b**)

Compound **20b** was synthesized as described for general method B by using K_2_CO_3_ (7.19 g, 52.0 mmol), KI (86 mg), 2-(3-chloropropyl)benzo[*d*]thiazole (**15b)**(1.10 g, 5.20 mmol), and 5-methoxy-2-(piperidin-4-yl)pyridine (1.00 g, 6.24 mmol) in an anhydrous acetonitrile (30 mL) solution. The crude product was purified by flash column chromatography to obtain pure **20b** as a sticky brown oil (880 mg, 46% yield). ^1^H NMR (400 MHz CDCl_3_) δ 8.20 (d, *J* = 2.9 Hz, 1H), 7.97–7.92 (m, 1H), 7.85–7.80 (m, 1H), 7.43 (ddd, *J* = 8.3, 7.2, 1.3 Hz, 1H), 7.33 (ddd, *J* = 8.2, 7.2, 1.2 Hz, 1H), 7.13 (dd, *J* = 8.7, 2.9 Hz, 1H), 7.07 (d, *J* = 8.6 Hz, 1H), 3.81 (s, 3H), 3.16 (t, *J* = 7.5 Hz, 2H), 3.06 (dt, *J* = 11.8, 3.2 Hz, 2H), 2.66 (tt, *J* = 11.7, 3.9 Hz, 1H), 2.51 (dd, *J* = 8.4, 6.3 Hz, 2H), 2.11 (td, *J* = 9.5, 3.2 Hz, 4H), 1.99–1.87 (m, 2H), 1.79 (qd, *J* = 12.3, 3.7 Hz, 2H). ^13^C NMR (101 MHz, CDCl_3_) δ: 171.97, 157.21, 154.08, 153.34, 136.41, 135.32, 125.97, 124.76, 122.61, 121.59, 121.43, 120.71, 57.74, 55.70, 54.08, 43.62, 32.29, 28.72, 26.94. The oxalate salt was precipitated from 2-propanol. Mp 181–182 °C. Anal. (C_21_H_25_N_3_OS•C_2_H_2_O_4_) C, H, N.

#### 6-Methyl-2-(3-(4-(pyridin-2-yl)piperidin-1-yl)propyl)benzo[*d*]thiazole (**20c**)

Compound **20c** was synthesized as described for general method B by using K_2_CO_3_ (4.10 g, 29.7 mmol), KI (49 mg), 2-(3-chloropropyl)-6-methylbenzo[*d*]thiazole (**18a**) (670 mg, 2.97 mmol), and 2-(piperidin-4-yl)pyridine (574 mg, 3.56 mmol) in an anhydrous acetonitrile (18 mL) solution. The crude product was purified by flash column chromatography to obtain pure **20c** as a sticky brown oil (630 mg, 60% yield). ^1^H NMR (400 MHz CDCl_3_) δ 8.50 (ddd, *J* = 5.0, 1.9, 0.9 Hz, 1H), 7.82 (d, *J* = 8.3 Hz, 1H), 7.62–7.57 (m, 2H), 7.23 (d, *J* = 1.7 Hz, 1H), 7.14 (dd, *J* = 7.9, 1.2 Hz, 1H), 7.09 (ddd, *J* = 7.4, 4.8, 1.1 Hz, 1H), 3.13 (t, *J* = 7.6 Hz, 2H), 3.06 (dt, *J* = 11.7, 3.0 Hz, 2H), 2.69 (tt, *J* = 12.1, 3.9 Hz, 1H), 2.48 (dd, *J* = 8.5, 6.3 Hz, 2H), 2.45 (s, 3H), 2.14–2.03 (m, *J* = 7.1, 4.6 Hz, 4H), 1.99–1.89 (m, 2H), 1.80 (qd, *J* = 12.3, 3.7 Hz, 2H). ^13^C NMR (101 MHz, CDCl_3_) δ: 170.94, 165.14, 151.41, 149.18, 136.61, 135.45, 134.78, 127.50, 122.06, 121.41, 121.38, 120.70, 57.79, 54.09, 44.70, 32.26, 32.08, 27.06, 21.56. The oxalate salt was precipitated from 2-propanol. Mp 151–152 °C. Anal. (C_21_H_25_N_3_S•2C_2_H_2_O_4_) C, H, N.

#### 7-Methoxy-2-(3-(4-(pyridin-2-yl)piperidin-1-yl)propyl)benzo[*d*]thiazole (**20d**)

Compound **20d** was synthesized as described for general method B by using K_2_CO_3_ (2.57 g, 18.6 mmol), KI (31 mg), 2-(3-chloropropyl)-7-methoxybenzo[*d*]thiazole (**18b**) (450 mg, 1.86 mmol), and 2-(piperidin-4-yl)pyridine (330 mg, 2.05 mmol) in an anhydrous acetonitrile (11 mL) solution. The crude product was purified by flash column chromatography to obtain pure **20d** as a brown oil (443 mg, 65% yield). ^1^H NMR (400 MHz CDCl_3_) δ 8.51 (ddd, *J* = 4.9, 1.9, 1.0 Hz, 1H), 7.64–7.56 (m, 2H), 7.38 (t, *J* = 8.1 Hz, 1H), 7.15 (dt, *J* = 8.0, 1.1 Hz, 1H), 7.09 (ddd, *J* = 7.5, 4.9, 1.1 Hz, 1H), 6.82–6.76 (m, 1H), 3.96 (s, 3H), 3.16 (t, *J* = 7.6 Hz, 2H), 3.06 (dt, *J* = 11.9, 3.1 Hz, 2H), 2.69 (tt, *J* = 12.0, 3.9 Hz, 1H), 2.49 (dd, *J* = 8.4, 6.3 Hz, 2H), 2.10 (qd, *J* = 8.7, 6.5 Hz, 4H), 1.98–1.90 (m, 2H), 1.81 (qd, *J* = 12.3, 3.8 Hz, 2H). ^13^C NMR (101 MHz, CDCl_3_) δ: 172.55, 165.18, 155.03, 154.30, 149.18, 136.57, 126.85, 123.85, 121.37, 120.69, 115.31, 104.85, 57.74, 55.96, 54.09, 44.71, 32.29, 32.09, 27.13. The oxalate salt was precipitated from 2-propanol. Mp 174–175 °C. Anal. (C_21_H_25_N_3_OS•C_2_H_2_O_4_) C, H, N.

#### 6-Methoxy-2-(3-(4-(pyridin-2-yl)piperidin-1-yl)propyl)benzo[*d*]thiazole (**20e**)

Compound **20e** was synthesized as described for general method B by using K_2_CO_3_ (2.34 g, 16.9 mmol), KI (28 mg), 2-(3-chloropropyl)-6-methoxybenzo[*d*]thiazole (**18c**) (409 mg, 1.69 mmol), and 2-(piperidin-4-yl)pyridine (300 mg, 1.86 mmol) in an anhydrous acetonitrile (10 mL) solution. The crude product was purified by flash column chromatography to obtain pure **20e** as a dark-brown oil (300 mg, 48% yield). ^1^H NMR (400 MHz CDCl_3_) δ 8.51 (ddd, *J* = 4.9, 1.9, 1.0 Hz, 1H), 7.82 (d, *J* = 8.9 Hz, 1H), 7.60 (td, *J* = 7.7, 1.8 Hz, 1H), 7.29 (d, *J* = 2.6 Hz, 1H), 7.15 (d, *J* = 7.9 Hz, 1H), 7.09 (ddd, *J* = 7.5, 4.8, 1.2 Hz, 1H), 7.03 (dd, *J* = 8.9, 2.5 Hz, 1H), 3.85 (s, 3H), 3.15–3.02 (m, 4H), 2.69 (tt, *J* = 12.1, 3.9 Hz, 1H), 2.49 (t, *J* = 7.3 Hz, 2H), 2.09 (td, *J* = 11.2, 5.0 Hz, 4H), 1.94 (d, *J* = 13.5 Hz, 2H), 1.88–1.69 (m, 2H). ^13^C NMR (101 MHz, CDCl_3_) δ: 169.07, 164.73, 157.45, 149.19, 147.76, 136.70, 136.53, 123.02, 121.47, 120.77, 115.14, 104.28, 57.58, 55.93, 55.89, 55.85, 53.86, 44.27, 32.04, 31.58, 26.55. The HCl salt was precipitated from 2-propanol. Mp 170–171 °C. Anal. (C_21_H_25_N_3_OS•C_2_H_2_O_4_) C, H, N.

### Radioligand Binding Assays

Binding at dopamine D_2_-like receptors was determined similarly to previously described methods.^2,48^ Membranes were prepared from HEK293 cells stably expressing human D_2L_R, D_3_R, or D_4_R grown in a 50:50 mix of DMEM and Ham’s F12 culture media, supplemented with 20 mM HEPES, 2 mM l-glutamine, 0.1 mM nonessential amino acids, 1× antibiotic/antimycotic, 10% heat-inactivated fetal bovine serum, and 200 μg/mL hygromycin (Life Technologies, Grand Island, New York) and kept in an incubator at 37 °C and 5% CO_2_. Upon reaching 80–90% confluence, cells were harvested using premixed Earle’s Balanced Salt Solution (EBSS) with 5 mM EDTA (Life Technologies) and centrifuged at 3000 rpm for 10 min at 21 °C. The supernatant was removed, and the pellet was resuspended in 10 mL of hypotonic lysis buffer (5 mM MgCl_2_ · 6 H_2_O, 5 mM Tris, pH 7.4 at 4 °C) and centrifuged at 14,500 rpm (∼25,000*g*) for 30 min at 4 °C. The pellet was then resuspended in fresh EBSS binding buffer made from 8.7 g/L Earle’s Balanced Salts without phenol red (US Biological, Salem, Massachusetts), 2.2 g/L sodium bicarbonate, pH to 7.4. A Bradford protein assay (Bio-Rad, Hercules, California) was used to determine the protein concentration, and membranes were diluted to 500 μg/mL and stored in a −80 °C freezer for later use.

Radioligand competition binding experiments were conducted using thawed membranes on test day, and each test compound was diluted into 10 half-log serial dilutions using 30% DMSO vehicle, starting from 1 mM or 100 μM concentration. Previously frozen membranes were diluted in fresh EBSS binding buffer to 200 μg/mL (for hD_2L_R or hD_3_R) or 400 μg/mL (for hD_4_R) for binding. Radioligand competition experiments were conducted in 96-well plates containing 300 μL of fresh EBSS binding buffer, 50 μL of diluted test compound, 100 μL of membranes (20 μg/well total protein for hD_2L_R and hD_3_R, and 50 μL of [^3^H]*N*-methylspiperone radioligand diluted in binding buffer (0.4 nM final concentration; PerkinElmer). Nonspecific binding was determined using 10 μM (+)-butaclamol (Sigma-Aldrich, St. Louis, Missouri), and total binding was determined with 30% DMSO vehicle. All compound dilutions were tested in triplicate and the reaction incubated for 1 h at RT. The reaction was terminated by filtration through PerkinElmer Uni-Filter-96 GF/C plates, presoaked for 1 h in 0.5% polyethylenimine, using a Brandel 96-Well Plates Harvester Manifold (Brandel Instruments, Gaithersburg, Maryland). The filters were washed (3 × 1 mL/well) in ice-cold binding buffer. PerkinElmer MicroScint 20 Scintillation Cocktail (65 μL) was added to each well, and filters were counted using a PerkinElmer MicroBeta Microplate Counter. IC_50_ values for each compound were determined from dose–response curves, and *K_i_* values were calculated using the Cheng–Prusoff equation.^47^ When a complete inhibition could not be achieved at the highest tested concentrations, *K_i_* values were extrapolated by constraining the bottom of the dose–response curves (= 0% residual specific binding) in the nonlinear regression analysis. These analyses were performed using GraphPad Prism versions 6.00–8.00 (GraphPad Software, San Diego, California). All results were rounded to three significant figures. *K_i_* values were determined from at least three independent experiments and are reported as means ± SEM.

### Functional Assays

#### cAMP Inhibition Assay

D_4_R- and D_2_R -mediated inhibition of forskolin-stimulated cAMP production was assayed using the PerkinElmer LANCE UltracAMP assay kit (PerkinElmer, Inc., Waltham, Massachusetts). CHO-K1 cells stably expressing the human D_2_R long isoform or D_4_R were maintained in Ham’s F12 supplemented with 10% fetal bovine serum, 100 U/mL penicillin, 100 μg/mL streptomycin, 800 μg/mL G418, and 300 μg/mL hygromycin at 37 °C, 5% CO_2_, and 90% humidity. Cells were seeded in 5 μL of Hank’s Balanced Salt Solution (with CaCl and MgCl_2_) with 5 mM HEPES buffer and 0.2 μM sodium metabisulfite at a density of 5000 cells/well in 384-well white plates. Compounds and forskolin were made in the same buffer. Immediately after plating, cells were treated with 2.5 μL of compound (at various concentrations) and 2.5 μL of forskolin and incubated at room temperature for 30 min. The final concentration of forskolin was 10 μM. When running the assay in antagonist mode, the EC_80_ of dopamine (10 nM) was added to the forskolin solution. Eu-cAMP tracer and ULight-anti-cAMP solutions were added as directed by the manufacturer, and cells were incubated for 2 h in the dark at room temperature, after which a TR-FRET signal was measured using a BMG Labtech PHERAstar FS (BMG Labtech, North Carolina). Values were normalized to a percentage of the control TR-FRET signal seen with a maximum concentration of dopamine for agonist mode assays and the EC_80_ of dopamine for antagonist mode assays. Data were collected in triplicate from at least three independent experiments. Data analysis and normalization were performed in GraphPad Prism 9 (GraphPad Software, California). First, raw data was fit using a log(agonist/antagonist) vs response – variable slope (four parameters) curve fit. The data were normalized to the percent maximum dopamine response (agonist mode) or the EC_80_ of dopamine (antagonist mode). The Hill coefficients of the concentration–response curves did not significantly differ from unity with the data fitting to a single-site model. Graphs are meaned concentration response curves from at least three independent experiments. Data in Table 2 was extracted from the meaned curves where *E*_max_/*I*_max_ are expressed as mean ± SEM and the potencies are expressed as mean [95% confidence interval]. Fold selectivities for D_4_R over the were also calculated and are presented in Table 2.

#### β-Arrestin Recruitment Assay

Assays were conducted with minor modifications, as previously published by our laboratory^2,19−23^ using the DiscoverX PathHunter technology (Eurofins DiscoverX, Fremont, California). Briefly, CHO-K1 cells stably expressing the human D_2_R long isoform, D_3_R, or D_4_R (Eurofins DiscoverX) were maintained in Ham’s F12 media supplemented with 10% fetal bovine serum, 100 U/mL penicillin, 100 μg/mL streptomycin, 800 μg/mL G418, and 300 μg/mL hygromycin at 37 °C, 5% CO_2_, and 90% humidity. The cells were seeded in 7.5 μL media at a density of 2625 cells/well in 384-well black, clear-bottom plates. The following day, the compounds were diluted in PBS with 0.2 μM sodium metabisulfite. The cells were treated with 16 concentrations of a compound in triplicate and incubated at 37 °C for 90 min. Tropix Gal-Screen Substrate (Applied Biosystems, Massachusetts) was diluted in Gal-Screen buffer A (Applied Biosystems) 1:25 and added to cells according to the manufacturer’s recommendations followed by a 30–45 min incubation at room temperature in the dark. Luminescence was measured on a Hamamatsu FDSS μCELL reader. Data was collected in triplicate and transferred to GraphPad Prism 9 where it was fit with a log(agonist/antagonist) vs response – variable slope (four parameters) curve fit. The data were normalized to the percent maximum dopamine response (agonist mode) or the EC_80_ of dopamine (antagonist mode). The Hill coefficients of the concentration–response curves did not significantly differ from unity, with the data fitting to a single-site model. Graphs are mean concentration response curves from at least three independent experiments. Data in Table 3 was extracted from the meaned curves where *E*_max_/*I*_max_ are expressed as mean ± SEM and the potencies are expressed as mean [95% confidence interval]. Fold selectivities for D_4_R over D_2_R and D_3_R were also calculated and are presented in Table 3.

#### Schild-Type Analysis – β-Arrestin Recruitment Assay

Schild-type analysis using the β-arrestin recruitment assay was conducted similarly except for compound preparation. Compounds were diluted in PBS with 0.2 μM sodium metabisulfite at eight concentrations ranging from 10 μM to 10 nM (final in assay concentrations) and a DMSO control. The compounds were added to the cells followed immediately by a dopamine concentration response curve and returned to the incubator at 37 °C for 90 min. The Tropic Gal-Screen substrate and buffer were prepared and added as previously described. All other aspects of the Schild-type analysis were identical to those of the β-arrestin recruitment assay procedure. Data was collected in triplicate and transferred to GraphPad Prism 9 (GraphPad Software, California) where it was fit with a log(agonist) vs response-variable slope (four parameters) curve fit. The data were normalized to the maximum dopamine/DMSO response. Graphs are meaned concentration response curves from at least three independent experiments. Schild-type plots were generated by plotting the log-scale compound concentration (*x*-axis) versus the log((*A′*/*A*) – 1) where *A′* is the EC_50_ of the dopamine curve obtained for each concentration of antagonist and *A* is the EC_50_ of dopamine in the DMSO control. Simple linear regression was performed in GraphPad Prism 9 where the slope and *x*-intercept indicate competitiveness and the affinity of the compound, respectively.

### Rat and Human Microsomal Stability Assays

Phase I metabolic stability assays were conducted using rat and human liver microsomes as previously described^41,49^ with minor modifications. In brief, the reactions were carried out with 100 mM potassium phosphate buffer, pH 7.4, in the presence of a NADPH regenerating system (1.3 mM NADPH, 3.3 mM glucose 6-phosphate, 3.3 mM MgCl_2_, 0.4 U/mL glucose-6-phosphate dehydrogenase, 50 μM sodium citrate). Negative controls without cofactors were assessed to determine the non-CYP-mediated metabolism. Positive controls for phase I metabolism (buprenorphine) were also evaluated. Compound disappearance was monitored over time using a liquid chromatography and tandem mass spectrometry (LC/MS) method. All reactions were performed in triplicate.

Chromatographic analysis was performed on a Dionex ultra high-performance LC system coupled with a Q Exactive Focus orbitrap mass spectrometer (Thermo Fisher Scientific Inc., Waltham, Massachusetts). Separation was achieved using an Agilent Eclipse Plus column (100 × 2.1 mm i.d; maintained at 35 °C) packed with a 1.8 μm C18 stationary phase. The mobile phase used was composed of 0.1% formic acid in acetonitrile and 0.1% formic acid in water with gradient elution, starting with 2.5% organic phase (from 0 to 2 min) linearly increasing to 99% (from 2 to 5.5 min) and re-equilibrating to 2.5% by 6.5 min. The total run time for each analyte was 6.5 min. Pumps were operated at a flow rate of 0.3 mL/min. The mass spectrometer controlled by Xcalibur software v.4.0.27.13 (Thermo Scientific) was operated with a HESI ion source in positive ionization mode. Compounds were identified in the full-scan mode (from *m*/*z* 50 to 750) by comparing *t* = 0 samples with *t* = 30 min and *t* = 60 min samples.

### Pharmacokinetics Study in Rats

Pharmacokinetic studies in Sprague–Dawley (SD) rats were conducted according to protocols approved by the Animal Care and Use Committee at Johns Hopkins University. SD rats obtained from Harlan were maintained on a 12 h light–dark cycle with ad libitum access to food and water. A test compound was administered via i.p. injection at a dose of 10 mg/kg (100% saline vehicle, 10 mL/kg volume). The rats were sacrificed at specified time points (0.25, 0.5, 1, 2, 4, and 6 h) post drug administration. For the collection of plasma and brain tissue, animals were euthanized with CO_2_ and blood samples were collected in heparinized microtubes by cardiac puncture. Brains were dissected and immediately flash-frozen (−80 °C). Blood samples were spun at 2000*g* for 15 min, and the plasma was removed and stored at −80 °C until analysis (as described below).

### Bioanalysis

Quantitation of **16f** was performed using liquid chromatography with tandem mass spectrometry (LC/MS-MS) methods. Briefly, calibration standards were prepared using respective tissue (naïve plasma and brain) with additions of the test compound. For quantifying the test compound in the pharmacokinetic samples, plasma samples (20 μL) were processed using a single liquid extraction method by addition of 100 μL of acetonitrile containing internal standard (losartan: 0.5 μM), followed by vortex-mixing for 30 s and then centrifugation at 10,000 × *g* for 10 min at 4 °C. Brain tissues were diluted 1:5 w/v with acetonitrile containing losartan (0.5 μm) and homogenized, followed by vortex-mixing and centrifugation at 10,000 × *g* for 10 min at 4 °C. A 50 μL aliquot of the supernatant was diluted with 50 μL of water and transferred to 250 μL polypropylene autosampler vials sealed with Teflon caps. 2 μL of the sample was injected into the LC/MS/MS system for analysis. Chromatographic analysis was performed using an Accela ultra high-performance system consisting of an analytical pump and an autosampler coupled with a TSQ Vantage mass spectrometer. Separation of the analyte was achieved at ambient temperature using an Agilent Eclipse Plus column (100 × 2.1 mm inner diameter) packed with a 1.8 μm C18 stationary phase. The mobile phase consisted of 0.1% formic acid in acetonitrile and 0.1% formic acid in water with gradient elution, starting with 10% organic phase (from 0 to 1 min) linearly increasing to 95% (from 1 to 2 min) and re-equilibrating to 10% by 3 min. The total run time for each analyte was 3.5 min. Pumps were operated at a flow rate of 0.3 mL/min. The [M + H]^+^ ion transitions of the test compound (**CAB-01–019**) (*m*/*z* 339.1638 → 121.0759, 176.0528) and losartan (IS) (*m*/*z* 423.1695 → 192.0808, 207.0914) were used. Plasma concentrations (nmol/mL) as well as brain tissue concentrations (nmol/g) were determined, and plots of mean plasma concentration versus time were constructed. Noncompartmental analysis modules in Phoenix WinNonlin version 7.0 (Certara USA, Inc., Princeton, New Jersey) were used to quantify exposures (AUC_0–*t*_) and half-life (*t*_1/2_).

### Operant Conditioning Experiments

#### Animals

Male Fischer 344 rats (100–130 days; Charles River, Wilmington, Massachusetts) were housed in a temperature-controlled vivarium on a 12 h reversed light/dark cycle (lights on at 6:00 PM). Rats were group-housed two per cage with water available *ad libitum* while food access was restricted to maintain consistent body weight during the experiment. Experimental sessions were conducted during the dark phase of the light/dark cycle. All procedures were performed in accordance with the High Point University Institutional Animal Care and Use Committee and the National Institutes of Health Guide for the Care and Use of Laboratory Animals (NIH Publication No. 80-23) revised in 1996.

#### Food-Maintained Responding

For experiments, rats were transferred to operant conditioning chambers (ENV-008CT; Med Associates, St. Albans, Vermont) enclosed in sound-attenuating cubicles (ENV-018; Med Associates). The front panel of the operant chambers contained two response levers (4 cm above the floor and 3 cm from each side wall), a cue light (3 cm above each of the two levers), and a food chute centered on the front wall (2 cm above the floor) that was connected to a food pellet dispenser (ENV-023; Med Associates) located behind the front wall and a tone generator to mask extraneous noise. Food-maintained responding was assessed using a multicomponent procedure consisting of three 30 min components separated by 4 min blackout periods between components. Responding was engendered and maintained by delivery of food pellets (45 mg; Noyes, Lancaster, New Hampshire; four, two, and one pellets for components 1, 2 and 3, respectively) under an FR3 schedule of reinforcement. Completion of the response requirement on the active lever extinguished lights, retracted both levers, and delivered food and was followed by a 20 s time-out (TO) period. After the TO, the lights were illuminated, the levers were extended, and the FR schedule was in effect. The presentation of **16f** doses (5, 15, 20, and 30 mg/kg, i.p.) and saline were randomly assigned and administered 15 min before the start of the session. The criterion for stable response was two consecutive sessions in which the total number of reinforcers did not vary by more than 10% from baseline levels.

### Cocaine Self-Administration

The operant apparatus was described above. For self-administration studies, a counterbalance arm was connected at the rear corner of the operant chamber, onto which a single channel swivel was mounted. The rat’s leash was attached to the swivel, and the catheter tubing was connected to the bottom port of the swivel. A motor-driven 20 mL syringe pump (PHM-100; Med Associates) was attached outside of the sound-attenuating chamber, and polyethylene tubing connected the needle on the syringe to the entry port of the swivel. A PC was used for session programming and data collection (Med Associates, Inc., East Fairfield, Vermont). For lever training, subjects were transferred to the operant chambers for daily experimental sessions and responding was engendered and maintained by delivery of food pellets (45 mg pellets; Noyes, Lancaster, NH) under an FR1 schedule of reinforcement that was gradually increased to FR3. The lever light was illuminated when the schedule was in effect. Completion of the response requirement on the active lever extinguished lights, retracted both levers, delivered food, and was followed by a 20 s time-out (TO) period during which all lights were off. After the TO, the lights were illuminated and the FR schedule was in effect. Sessions lasted 30 min or until 50 food pellets were delivered. The criterion for stable response was five consecutive sessions in which the total number of reinforcers did not vary by more than 20% from control levels. Responses on the inactive lever were recorded but had no scheduled consequences.

#### Intravenous Jugular Surgery

After operant responding was acquired and maintained by food, subjects were surgically implanted with a venous catheter inserted into the right jugular vein following administration of ketamine (90 mg/kg; i.p.) and xylazine (5 mg/kg; i.p.) for anesthesia, as described previously.^50−52^ Catheters were anchored to the muscle near the point of entry into the vein. The distal end of the catheter was guided subcutaneously to exit above the scapulae through a Teflon shoulder harness. The harness provided a point of attachment for a spring leash connected to a single-channel fluid swivel at the opposing end. The catheter was threaded through the leash and attached to the swivel. The other end of the swivel was connected to a syringe (for saline and drug delivery) mounted on a syringe pump. Rats were administered penicillin G procaine (75,000 units in 0.25 mL, i.m.) and allowed a minimum of 5 days to recover before self-administration studies were initiated. Following surgery, rats received hourly infusions of heparinized 0.9% bacteriostatic saline (1.7 U/mL; 200 μL/h) using a computer-controlled motor-driven syringe pump in the home cage vivarium. The health of the rats was monitored daily by the experimenters and weekly by an institutional veterinarian per the guidelines issued by the High Point University Institutional Animal Care and Use Committee and the National Institutes of Health. Infusions of propofol (6 mg/kg; (iv) were administered to assess the catheter patency, as needed.

Responding was maintained under an FR3:20 s TO of three 1 h components. Subjects were allowed to self-administer cocaine iv (166, 83, and 41.5 mg/infusion). Each dose was available during a different component, and the doses were presented in descending order. The infusion volume for the first component was 400 μL infused over 12 s, and the volumes for the successive components were 200 μL for component 2 (infused over 6 s) and 100 μL for component 3 (infused over 3 s). Before each component, a 10 min blackout was followed by a priming infusion of the dose to be administered in the succeeding component. After an additional 10 min blackout period, the lever was activated and the cue light above the lever was illuminated. The start of each session was indicated by the illumination of the house light, stimulus light above the active lever, and the extension of both levers. Upon completion of the response requirement, a drug infusion was delivered, the lever light was extinguished, a tone was generated, and the house light was illuminated. During the 20 s TO after the infusion, responses on the lever were recorded but had no scheduled consequence. A minimum of 3 days of stable responding (less than 10% variation in the number of infusions) at FR3 in all components was required before administration of compounds was initiated.

#### Effects of **16f** on Cocaine Self-Administration

Rats were transferred to the operant chambers for self-administration sessions. Before each session, the swivel and catheter were flushed with 500 μL of heparinized saline before connecting the catheter to the syringe via a 20 ga Leur hub and 28 ga male connector. Completion of the response requirement on the active lever extinguished lights, retracted both levers, and delivered food,and was followed by 20 s TO. After the TO, lights were illuminated, levers extended, and the FR schedule was in effect.^50−54^

After a minimum of 5 days of stable responding (defined as consecutive sessions in which the total number of infusions did not vary by more than 20% from the mean of previous sessions), saline vehicle and **16f** (5, 15, 20, and 30 mg/kg, i.p.) were tested. Dose order was randomly assigned for each subject. **16f** and saline were administered 15 min before the first component.

## Supporting Information Available

Elemental analysis for all final compounds results; library of **16f** analogues for virtual docking; computational Analysis; molecular docking analysis, CNS-MPO value and method of calculation for **16f**; and HPLC and MS traces of **16f** (PDF)
SMILES data (CSV)

## Abbreviations

CDCl_3_: deuterated chloroform
CD_3_OD: deuterated methanol
CMA: chloroform/methanol/ammonium hydroxide
5% CMA: (95% chloroform, 4% methanol, 1% ammonium hydroxide)
EtOAc: ethyl acetate
DA: dopamine
D_2_R: dopamine D_2_ receptor
D_3_R: dopamine D_3_ receptor
D_4_R: dopamine D_4_ receptor
NMR: nuclear magnetic resonance
RT: room temperature
SAR: structure activity relationship.

## References

[ref1] Beaulieu J. M.; Gainetdinov R. R. The physiology, signaling, and pharmacology of dopamine receptors. Pharmacol. Rev. 2011, 63, 182–217. 10.1124/pr.110.002642. 21303898

[ref2] Keck T. M.; Free R. B.; Day M. M.; Brown S. L.; Maddaluna M. S.; Fountain G.; Cooper C.; Fallon B.; Holmes M.; Stang C. T.; Burkhardt R.; Bonifazi A.; Ellenberger M. P.; Newman A. H.; Sibley D. R.; Wu C.; Boateng C. A. Dopamine D4 Receptor-Selective Compounds Reveal Structure-Activity Relationships that Engender Agonist Efficacy. J. Med. Chem. 2019, 62, 3722–3740. 10.1021/acs.jmedchem.9b00231. 30883109 PMC6466480

[ref3] Tomlinson A.; Grayson B.; Marsh S.; Hayward A.; Marshall K. M.; Neill J. C. Putative therapeutic targets for symptom subtypes of adult ADHD: D4 receptor agonism and COMT inhibition improve attention and response inhibition in a novel translational animal model. Eur. Neuropsychopharmacol. 2015, 25, 454–467. 10.1016/j.euroneuro.2014.11.016. 25799918

[ref4] Ferre S.; Belcher A. M.; Bonaventura J.; Quiroz C.; Sanchez-Soto M.; Casado-Anguera V.; Cai N. S.; Moreno E.; Boateng C. A.; Keck T. M.; Floran B.; Earley C. J.; Ciruela F.; Casado V.; Rubinstein M.; Volkow N. D. Functional and pharmacological role of the dopamine D_4_ receptor and its polymorphic variants. Front. Endocrinol. (Lausanne) 2022, 13, 1014678 10.3389/fendo.2022.1014678. 36267569 PMC9578002

[ref5] Botticelli L.; Micioni Di Bonaventura E.; Del Bello F.; Giorgioni G.; Piergentili A.; Romano A.; Quaglia W.; Cifani C.; Micioni Di Bonaventura M. V. Underlying Susceptibility to Eating Disorders and Drug Abuse: Genetic and Pharmacological Aspects of Dopamine D4 Receptors. Nutrients 2020, 12, 2288 10.3390/nu12082288. 32751662 PMC7468707

[ref6] Giorgioni G.; Del Bello F.; Pavletic P.; Quaglia W.; Botticelli L.; Cifani C.; Micioni Di Bonaventura E.; Micioni Di Bonaventura M. V.; Piergentili A. Recent findings leading to the discovery of selective dopamine D4 receptor ligands for the treatment of widespread diseases. Eur. J. Med. Chem. 2021, 212, 113141 10.1016/j.ejmech.2020.113141. 33422983

[ref7] Lindsley C. W.; Hopkins C. R. Return of D4 Dopamine Receptor Antagonists in Drug Discovery. J. Med. Chem. 2017, 60, 7233–7243. 10.1021/acs.jmedchem.7b00151. 28489950

[ref8] Bernaerts P.; Tirelli E. Facilitatory effect of the dopamine D4 receptor agonist PD168,077 on memory consolidation of an inhibitory avoidance learned response in C57BL/6J mice. Behav. Brain Res. 2003, 142, 41–52. 10.1016/S0166-4328(02)00371-6. 12798264

[ref9] Nakazawa S.; Murai T.; Miyauchi M.; Kotani M.; Ikeda K. Behavioral and neurophysiological effects of Ro 10–5824, a dopamine D4 receptor partial agonist, in common marmosets. Psychopharmacology (Berlin) 2015, 232, 3287–3295. 10.1007/s00213-015-3978-y. 26041337

[ref10] Woolley M. L.; Waters K. A.; Reavill C.; Bull S.; Lacroix L. P.; Martyn A. J.; Hutcheson D. M.; Valerio E.; Bate S.; Jones D. N.; Dawson L. A. Selective dopamine D4 receptor agonist (A-412997) improves cognitive performance and stimulates motor activity without influencing reward-related behaviour in rat. Behav. Pharmacol. 2008, 19, 765–776. 10.1097/FBP.0b013e32831c3b06. 19020411

[ref11] Sood P.; Idris N. F.; Cole S.; Grayson B.; Neill J. C.; Young A. M. PD168077, a D_4_ receptor agonist, reverses object recognition deficits in rats: potential role for D_4_ receptor mechanisms in improving cognitive dysfunction in schizophrenia. J. Psychopharmacol. 2011, 25, 792–800. 10.1177/0269881110387840. 21088042

[ref12] Browman K. E.; Curzon P.; Pan J. B.; Molesky A. L.; Komater V. A.; Decker M. W.; Brioni J. D.; Moreland R. B.; Fox G. B. A-412997, a selective dopamine D4 agonist, improves cognitive performance in rats. Pharmacol., Biochem. Behav. 2005, 82, 148–155. 10.1016/j.pbb.2005.08.002. 16154186

[ref13] Rondou P.; Haegeman G.; Van Craenenbroeck K. The dopamine D4 receptor: biochemical and signalling properties. Cell. Mol. Life Sci. 2010, 67, 1971–1986. 10.1007/s00018-010-0293-y. 20165900 PMC11115718

[ref14] Di Ciano P.; Grandy D. K.; Le Foll B. Dopamine D4 receptors in psychostimulant addiction. Adv. Pharmacol. 2014, 69, 301–321. 10.1016/B978-0-12-420118-7.00008-1. 24484981 PMC4410854

[ref15] Bergman J.; Rheingold C. G. Dopamine D_4_ Receptor Antagonists for the Treatment of Cocaine Use Disorders. CNS Neurol. Disord. Drug Targets 2015, 14, 707–715. 10.2174/1871527314666150529132723. 26022267

[ref16] Sebastianutto I.; Maslava N.; Hopkins C. R.; Cenci M. A. Validation of an improved scale for rating l-DOPA-induced dyskinesia in the mouse and effects of specific dopamine receptor antagonists. Neurobiol. Dis. 2016, 96, 156–170. 10.1016/j.nbd.2016.09.001. 27597526

[ref17] Tolentino K. T.; Mashinson V.; Vadukoot A. K.; Hopkins C. R. Discovery and characterization of benzyloxy piperidine based dopamine 4 receptor antagonists. Bioorg. Med. Chem. Lett. 2022, 61, 128615 10.1016/j.bmcl.2022.128615. 35151866 PMC8966054

[ref18] Tallman J. F.; Primus R. J.; Brodbeck R.; Cornfield L.; Meade R.; Woodruff K.; Ross P.; Thurkauf A.; Gallager D. W. I. NGD 94–1: identification of a novel, high-affinity antagonist at the human dopamine D4 receptor. J. Pharmacol. Exp. Ther. 1997, 282, 1011–1019. 9262370

[ref19] Kim A.; Di Ciano P.; Pushparaj A.; Leca J.; Le Foll B. The effects of dopamine D4 receptor ligands on operant alcohol self-administration and cue- and stress-induced reinstatement in rats. Eur. J. Pharmacol. 2020, 867, 172838 10.1016/j.ejphar.2019.172838. 31794709

[ref20] Belliotti T. R.; Wustrow D. J.; Brink W. A.; Zoski K. T.; Shih Y. H.; Whetzel S. Z.; Georgic L. M.; Corbin A. E.; Akunne H. C.; Heffner T. G.; Pugsley T. A.; Wise L. D. A series of 6- and 7-piperazinyl- and -piperidinylmethylbenzoxazinones with dopamine D4 antagonist activity: discovery of a potential atypical antipsychotic agent. J. Med. Chem. 1999, 42, 5181–5187. 10.1021/jm990277d. 10602703

[ref21] Patel S.; Freedman S.; Chapman K. L.; Emms F.; Fletcher A. E.; Knowles M.; Marwood R.; McAllister G.; Myers J.; Curtis N.; Kulagowski J. J.; Leeson P. D.; Ridgill M.; Graham M.; Matheson S.; Rathbone D.; Watt A. P.; Bristow L. J.; Rupniak N. M.; Baskin E.; Lynch J. J.; Ragan C. I. Biological profile of L-745,870, a selective antagonist with high affinity for the dopamine D4 receptor. J. Pharmacol. Exp. Ther. 1997, 283, 636–647. 9353380

[ref22] Belliotti T. R.; Brink W. A.; Kesten S. R.; Rubin J. R.; Wustrow D. J.; Zoski K. T.; Whetzel S. Z.; Corbin A. E.; Pugsley T. A.; Heffner T. G.; Wise L. D. Isoindolinone enantiomers having affinity for the dopamine D4 receptor. Bioorg. Med. Chem. Lett. 1998, 8, 1499–1502. 10.1016/S0960-894X(98)00252-2. 9873377

[ref23] Bristow L. J.; Collinson N.; Cook G. P.; Curtis N.; Freedman S. B.; Kulagowski J. J.; Leeson P. D.; Patel S.; Ragan C. I.; Ridgill M.; Saywell K. L.; Tricklebank M. D. L-745,870, a subtype selective dopamine D4 receptor antagonist, does not exhibit a neuroleptic-like profile in rodent behavioral tests. J. Pharmacol. Exp. Ther. 1997, 283, 1256–1263. 9400001

[ref24] Bristow L. J.; Kramer M. S.; Kulagowski J.; Patel S.; Ragan C. I.; Seabrook G. R. Schizophrenia and L-745, 870, a novel dopamine D4 receptor antagonist. Trends Pharmacol. Sci. 1997, 18, 186–188. 10.1016/S0165-6147(97)90618-0. 9226994

[ref25] Pavletic P.; Semeano A.; Yano H.; Bonifazi A.; Giorgioni G.; Piergentili A.; Quaglia W.; Sabbieti M. G.; Agas D.; Santoni G.; Pallini R.; Ricci-Vitiani L.; Sabato E.; Vistoli G.; Del Bello F. Highly Potent and Selective Dopamine D4 Receptor Antagonists Potentially Useful for the Treatment of Glioblastoma. J. Med. Chem. 2022, 65, 12124–12139. 10.1021/acs.jmedchem.2c00840. 36098685 PMC9511495

[ref26] Mammoli V.; Bonifazi A.; Dal Ben D.; Giannella M.; Giorgioni G.; Piergentili A.; Pigini M.; Quaglia W.; Thomas A.; Newman A. H.; Ferre S.; Sanchez-Soto M.; Keck T. M.; Del Bello F. A Novel Class of Dopamine D_4_ Receptor Ligands Bearing an Imidazoline Nucleus. ChemMedChem 2016, 11, 1819–1828. 10.1002/cmdc.201600022. 26990230 PMC4993638

[ref27] Rowley M.; Broughton H. B.; Collins I.; Baker R.; Emms F.; Marwood R.; Patel S.; Patel S.; Ragan C. I.; Freedman S. B.; Leeson P. D. 5-(4-Chlorophenyl)-4-methyl-3-(1-(2-phenylethyl)piperidin-4-yl)isoxazole: a potent, selective antagonist at human cloned dopamine D4 receptors. J. Med. Chem. 1996, 39, 1943–1945. 10.1021/jm960072u. 8642551

[ref28] Wang S.; Wacker D.; Levit A.; Che T.; Betz R. M.; McCorvy J. D.; Venkatakrishnan A. J.; Huang X. P.; Dror R. O.; Shoichet B. K.; Roth B. L. D4 dopamine receptor high-resolution structures enable the discovery of selective agonists. Science 2017, 358, 381–386. 10.1126/science.aan5468. 29051383 PMC5856174

[ref29] Witt J. O.; McCollum A. L.; Hurtado M. A.; Huseman E. D.; Jeffries D. E.; Temple K. J.; Plumley H. C.; Blobaum A. L.; Lindsley C. W.; Hopkins C. R. Synthesis and characterization of a series of chiral alkoxymethyl morpholine analogs as dopamine receptor 4 (D4R) antagonists. Bioorg. Med. Chem. Lett. 2016, 26, 2481–2488. 10.1016/j.bmcl.2016.03.102. 27080176 PMC5361409

[ref30] Van Tol H. H.; Bunzow J. R.; Guan H. C.; Sunahara R. K.; Seeman P.; Niznik H. B.; Civelli O. Cloning of the gene for a human dopamine D4 receptor with high affinity for the antipsychotic clozapine. Nature 1991, 350, 610–614. 10.1038/350610a0. 1840645

[ref31] Sampson D.; Zhu X. Y.; Eyunni S. V.; Etukala J. R.; Ofori E.; Bricker B.; Lamango N. S.; Setola V.; Roth B. L.; Ablordeppey S. Y. Identification of a new selective dopamine D4 receptor ligand. Bioorg. Med. Chem. 2014, 22, 3105–3114. 10.1016/j.bmc.2014.04.026. 24800940 PMC4096627

[ref32] Cowart M. D. L. S. P.; Nelson S. L.; Stewart A. O. Fused Bicyclic Aromatic Compounds that are Useful in Treating Sexual Dysfunction. U.S Patent 2006/0172995. August 3, 2006.

[ref33] Sastry G. M.; Adzhigirey M.; Day T.; Annabhimoju R.; Sherman W. Protein and ligand preparation: parameters, protocols, and influence on virtual screening enrichments. J. Comput.-Aided Mol. Des. 2013, 27, 221–234. 10.1007/s10822-013-9644-8. 23579614

[ref34] Harder E.; Damm W.; Maple J.; Wu C.; Reboul M.; Xiang J. Y.; Wang L.; Lupyan D.; Dahlgren M. K.; Knight J. L.; Kaus J. W.; Cerutti D. S.; Krilov G.; Jorgensen W. L.; Abel R.; Friesner R. A. OPLS3: A Force Field Providing Broad Coverage of Drug-like Small Molecules and Proteins. J. Chem. Theory Comput. 2016, 12, 281–296. 10.1021/acs.jctc.5b00864. 26584231

[ref35] Banala A. K.; Levy B. A.; Khatri S. S.; Furman C. A.; Roof R. A.; Mishra Y.; Griffin S. A.; Sibley D. R.; Luedtke R. R.; Newman A. H. N-(3-fluoro-4-(4-(2-methoxy or 2,3-dichlorophenyl)piperazine-1-yl)butyl)arylcarboxamides as selective dopamine D3 receptor ligands: critical role of the carboxamide linker for D3 receptor selectivity. J. Med. Chem. 2011, 54, 3581–3594. 10.1021/jm200288r. 21495689 PMC3100590

[ref36] Cha M. Y.; Choi B. C.; Kang K. H.; Pae A. N.; Choi K. I.; Cho Y. S.; Koh H. Y.; Lee H. Y.; Jung D.; Kong J. Y. Design and synthesis of a piperazinylalkylisoxazole library for subtype selective dopamine receptor ligands. Bioorg. Med. Chem. Lett. 2002, 12, 1327–1330. 10.1016/S0960-894X(02)00179-8. 11992769

[ref37] Stewart A. O.; Cowart M. D.; Moreland R. B.; Latshaw S. P.; Matulenko M. A.; Bhatia P. A.; Wang X.; Daanen J. F.; Nelson S. L.; Terranova M. A.; Namovic M. T.; Donnelly-Roberts D. L.; Miller L. N.; Nakane M.; Sullivan J. P.; Brioni J. D. Dopamine D4 ligands and models of receptor activation: 2-(4-pyridin-2-ylpiperazin-1-ylmethyl)-1H-benzimidazole and related heteroarylmethylarylpiperazines exhibit a substituent effect responsible for additional efficacy tuning. J. Med. Chem. 2004, 47, 2348–2355. 10.1021/jm0305669. 15084133

[ref38] Lober S.; Hubner H.; Utz W.; Gmeiner P. Rationally based efficacy tuning of selective dopamine d4 receptor ligands leading to the complete antagonist 2-[4-(4-chlorophenyl)piperazin-1-ylmethyl]pyrazolo[1,5-a]pyridine (FAUC 213). J. Med. Chem. 2001, 44, 2691–2694. 10.1021/jm015522j. 11495580

[ref39] Besnard J.; Ruda G. F.; Setola V.; Abecassis K.; Rodriguiz R. M.; Huang X. P.; Norval S.; Sassano M. F.; Shin A. I.; Webster L. A.; Simeons F. R.; Stojanovski L.; Prat A.; Seidah N. G.; Constam D. B.; Bickerton G. R.; Read K. D.; Wetsel W. C.; Gilbert I. H.; Roth B. L.; Hopkins A. L. Automated design of ligands to polypharmacological profiles. Nature 2012, 492, 215–220. 10.1038/nature11691. 23235874 PMC3653568

[ref40] Wager T. T.; Hou X.; Verhoest P. R.; Villalobos A. Central Nervous System Multiparameter Optimization Desirability: Application in Drug Discovery. ACS Chem. Neurosci. 2016, 7, 767–775. 10.1021/acschemneuro.6b00029. 26991242

[ref41] Battiti F. O.; Cemaj S. L.; Guerrero A. M.; Shaik A. B.; Lam J.; Rais R.; Slusher B. S.; Deschamps J. R.; Imler G. H.; Newman A. H.; Bonifazi A. The Significance of Chirality in Drug Design and Synthesis of Bitopic Ligands as D3 Receptor (D3R) Selective Agonists. J. Med. Chem. 2019, 62, 6287–6314. 10.1021/acs.jmedchem.9b00702. 31257877 PMC8272914

[ref42] Sholler D. J.; Stutz S. J.; Fox R. G.; Boone E. L.; Wang Q.; Rice K. C.; Moeller F. G.; Anastasio N. C.; Cunningham K. A. The 5-HT(2A) Receptor (5-HT(2A)R) Regulates Impulsive Action and Cocaine Cue Reactivity in Male Sprague-Dawley Rats. J. Pharmacol. Exp. Ther. 2019, 368, 41–49. 10.1124/jpet.118.251199. 30373886 PMC6290084

[ref43] Pockros L. A.; Pentkowski N. S.; Swinford S. E.; Neisewander J. L. Blockade of 5-HT2A receptors in the medial prefrontal cortex attenuates reinstatement of cue-elicited cocaine-seeking behavior in rats. Psychopharmacology (Berlin) 2011, 213, 307–320. 10.1007/s00213-010-2071-9. 21079923 PMC3072217

[ref44] Nic Dhonnchadha B. A.; Fox R. G.; Stutz S. J.; Rice K. C.; Cunningham K. A. Blockade of the serotonin 5-HT2A receptor suppresses cue-evoked reinstatement of cocaine-seeking behavior in a rat self-administration model. Behav. Neurosci. 2009, 123, 382–396. 10.1037/a0014592. 19331461 PMC3830454

[ref45] Doly S.; Quentin E.; Eddine R.; Tolu S.; Fernandez S. P.; Bertran-Gonzalez J.; Valjent E.; Belmer A.; Vinals X.; Callebert J.; Faure P.; Meye F. J.; Herve D.; Robledo P.; Mameli M.; Launay J. M.; Maldonado R.; Maroteaux L. Serotonin 2B Receptors in Mesoaccumbens Dopamine Pathway Regulate Cocaine Responses. J. Neurosci. 2017, 37, 10372–10388. 10.1523/JNEUROSCI.1354-17.2017. 28935766 PMC6596631

[ref46] Cathala A.; Devroye C.; Robert E.; Vallee M.; Revest J. M.; Artigas F.; Spampinato U. Serotonin2B receptor blockade in the rat dorsal raphe nucleus suppresses cocaine-induced hyperlocomotion through an opposite control of mesocortical and mesoaccumbens dopamine pathways. Neuropharmacology 2020, 180, 108309 10.1016/j.neuropharm.2020.108309. 32956675

[ref47] Czoty P. W.; McCabe C.; Nader M. A. Effects of the 5-HT(1A) agonist (±)-8-hydroxy-2-(di-n-propylamino)tetralin (8-OH-DPAT) on cocaine choice in cynomolgus monkeys. Behav. Pharmacol. 2005, 16, 187–191. 10.1097/00008877-200505000-00008. 15864074

[ref48] Boateng C. A.; Bakare O. M.; Zhan J.; Banala A. K.; Burzynski C.; Pommier E.; Keck T. M.; Donthamsetti P.; Javitch J. A.; Rais R.; Slusher B. S.; Xi Z. X.; Newman A. H. High Affinity Dopamine D3 Receptor (D3R)-Selective Antagonists Attenuate Heroin Self-Administration in Wild-Type but not D3R Knockout Mice. J. Med. Chem. 2015, 58, 6195–6213. 10.1021/acs.jmedchem.5b00776. 26203768 PMC4937837

[ref49] Kumar V.; Bonifazi A.; Ellenberger M. P.; Keck T. M.; Pommier E.; Rais R.; Slusher B. S.; Gardner E.; You Z. B.; Xi Z. X.; Newman A. H. Highly Selective Dopamine D3 Receptor (D3R) Antagonists and Partial Agonists Based on Eticlopride and the D3R Crystal Structure: New Leads for Opioid Dependence Treatment. J. Med. Chem. 2016, 59, 7634–7650. 10.1021/acs.jmedchem.6b00860. 27508895 PMC5001167

[ref50] Pattison L. P.; McIntosh S.; Sexton T.; Childers S. R.; Hemby S. E. Changes in dopamine transporter binding in nucleus accumbens following chronic self-administration cocaine: heroin combinations. Synapse 2014, 68, 437–444. 10.1002/syn.21755. 24916769 PMC4687011

[ref51] Pattison L. P.; McIntosh S.; Budygin E. A.; Hemby S. E. Differential regulation of accumbal dopamine transmission in rats following cocaine, heroin and speedball self-administration. J. Neurochem. 2012, 122, 138–146. 10.1111/j.1471-4159.2012.07738.x. 22443145 PMC3843350

[ref52] McIntosh S.; Sexton T.; Pattison L. P.; Childers S. R.; Hemby S. E. Increased Sensitivity to Cocaine Self-Administration in HIV-1 Transgenic Rats is Associated with Changes in Striatal Dopamine Transporter Binding. J. Neuroimmune Pharmacol. 2015, 10, 493–505. 10.1007/s11481-015-9594-0. 25749646 PMC4701048

[ref53] Hemby S. E.; Co C.; Dworkin S. I.; Smith J. E. Synergistic elevations in nucleus accumbens extracellular dopamine concentrations during self-administration of cocaine/heroin combinations (Speedball) in rats. J. Pharmacol. Exp. Ther. 1999, 288, 274–280. 9862781

[ref54] Hemby S. E.; Smith J. E.; Dworkin S. I. The effects of eticlopride and naltrexone on responding maintained by food, cocaine, heroin and cocaine/heroin combinations in rats. J. Pharmacol. Exp. Ther. 1996, 277, 1247–1258. 8667185

