# Supplementary material for: Pharmacology and Therapeutic Potential of Benzothiazole Analogues for Cocaine Use Disorder
Source: J Med Chem. 2023 Aug 30;66(17):12141–62. doi: 10.1021/acs.jmedchem.3c00734 (PMC10510399; doi:10.1021/acs.jmedchem.3c00734)
Supplement: Supplementary file 1 — jm3c00734_si_001.pdf [file jm3c00734_si_001.pdf]

Supporting Information

Pharmacology and Therapeutic Potential of Benzothiazole Analogues for Cocaine Use Disorder

Comfort A. Boateng,[†,#,] Ashley N. Nilson,[≠#] Rebekah Placide,[†] Mimi L. Pham,[†] Franziska M. Jakobs,[†] Noelia Boldizsar,[≠] Scot McIntosh,[†] Leia S. Stallings,[†] Ivana V. Korankyi,[†] Shreya Kelshikar,[+] Nisha Shah,[+] Diandra Panasis,[+] Abigail Muccilli,[+] Maria Ladik,[+] Brianna Maslonka,[+] Connor McBride,[+] Moises Ximello Sanchez,[+] Ebrar Akca,[+] Mohammad Alkhatib,[+] Julianna Saez,[+] Catherine Nguyen,[+] Emily Kurtyan,[+] Jacquelyn DePierro,[+] Raymond Crowthers,[+] Dylan Brunt,[+] Alessandro Bonifazi,[^] Amy Hauck Newman,[^] Rana Rais,[‡] Barbara S. Slusher,[‡] R. Benjamin Free,[≠] David R. Sibley,[≠] Kent D. Stewart,[†] Chun Wu,[+] Scott E. Hemby,[†] Thomas M. Keck[+,*]*

[†]Department of Basic Pharmaceutical Sciences, Fred Wilson School of Pharmacy, High Point University, One University Parkway, High Point, North Carolina, 27268, United States

[+]Department of Chemistry & Biochemistry, Department of Biological & Biomedical Sciences, College of Science and Mathematics, Rowan University, 201 Mullica Hill Road, Glassboro, New Jersey, 08028, United States

[^]Medicinal Chemistry Section, Molecular Targets and Medications Discovery Branch, National Institute on Drug Abuse-Intramural Research Program, National Institutes of Health, 333 Cassell Drive, Baltimore, Maryland, 21224, United States

[‡]Department of Neurology, Johns Hopkins Drug Discovery, The Johns Hopkins University School of Medicine, 855 N. Wolfe Street, Baltimore, MD 21205, United States

[≠]Molecular Neuropharmacology Section, National Institute of Neurological Disorders and Stroke-Intramural Research Program, National Institutes of Health, Bethesda, Maryland, 20892, United States

[#] Equally Contributing Authors, * Corresponding Authors

Supporting information Contents:

Content	Page
Table S1: Microanalysis data of all compounds	S4
Figure S1. Library of 16f analogues for virtual docking	S5
Table S2. Computational Analysis	S6
Figure S2. Molecular docking of 16f at D_4R	S7
Table S3: CNS-MPO value and method of calculation for 16f	S8
Figure S3. HPLC and MS traces of 16f.	S9

Table S1. Microanalysis data.

Compound	C	H	N	C	H	N
	Calculated			Found		
**16a**	52.14	5.79	16.08	52.41	6.02	16.08
**16b**	49.14	6.12	11.30	49.14	6.19	11.46
**16c**	50.98	6.23	11.18	50.61	6.37	11.24
**16d**	55.40	5.28	11.75	55.57	5.36	11.76
**16e**	48.91	5.71	15.34	48.70	6.02	15.78
**16f**	48.19	6.16	11.73	48.06	5.94	11.80
**19a**	48.82	6.13	11.36	48.69	6.23	11.36
**19b**	47.61	6.17	10.97	47.58	5.99	11.10
**19c**	48.29	6.23	11.05	48.25	6.28	11.25
**19d**	46.47	5.17	11.34	46.45	5.13	11.40
**19e**	44.31	5.38	10.89	44.41	5.39	10.90
**19f**	47.25	5.15	11.56	46.88	5.07	11.51
**20a**	61.84	6.66	8.51	61.81	7.06	8.32
**20b**	60.38	5.95	9.18	60.13	6.00	9.04
**20c**	56.53	5.73	7.44	56.49	5.50	7.90
**20d**	59.79	5.99	8.88	59.79	6.00	9.09
**20e**	60.38	5.95	9.18	60.29	5.98	9.00

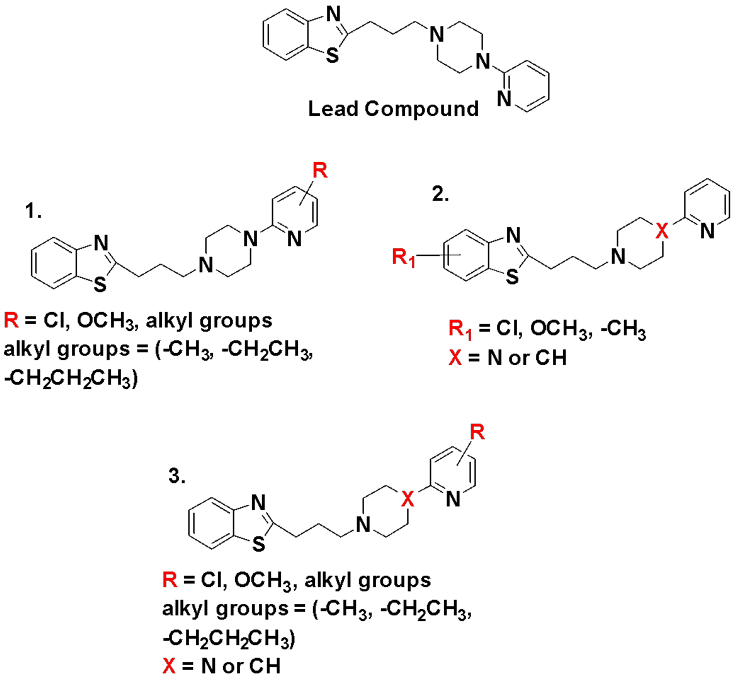

Figure S1. Library of **16f** analogues created for virtual screening.

Table S2. Docking analysis of proposed Series 2 ligands.

Compound	Docking Score (kcal/mol)
**16f**	-9.273
**19a**	-8.259
**19b**	-9.268
**19c**	-8.794
**19d**	-8.602
**19e**	-8.240
**19f**	-8.371
**20a**	-9.304
**20b**	-9.608
**20c**	-9.152
**20d**	-8.980
**20e**	-9.264

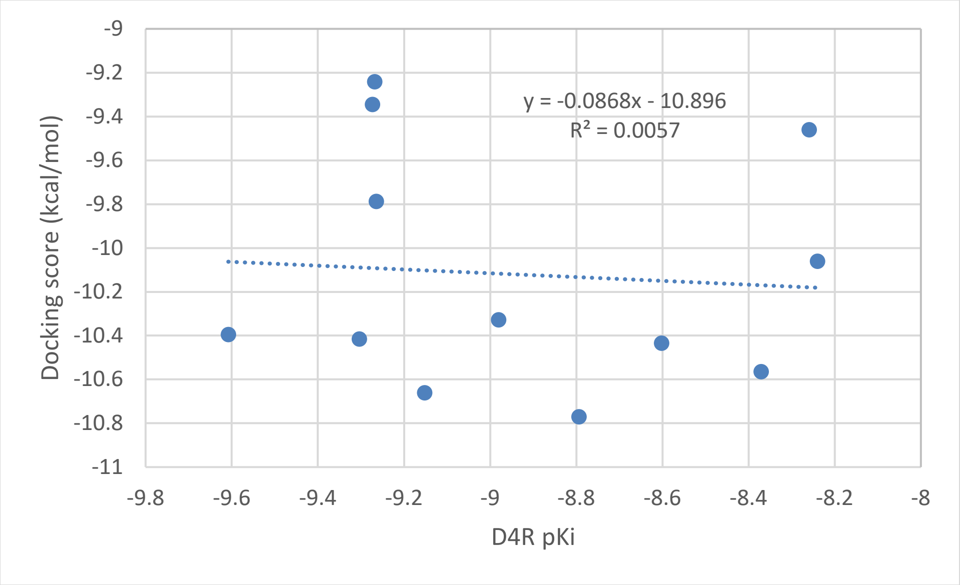

Overall correlation between docking score and experimentally determined compound affinity at D_4R.

Figure S2. Docking of Compound **16f** using the crystal structure of D_4R. Interaction diagrams (left) and 3D representations with interacting residues (right).

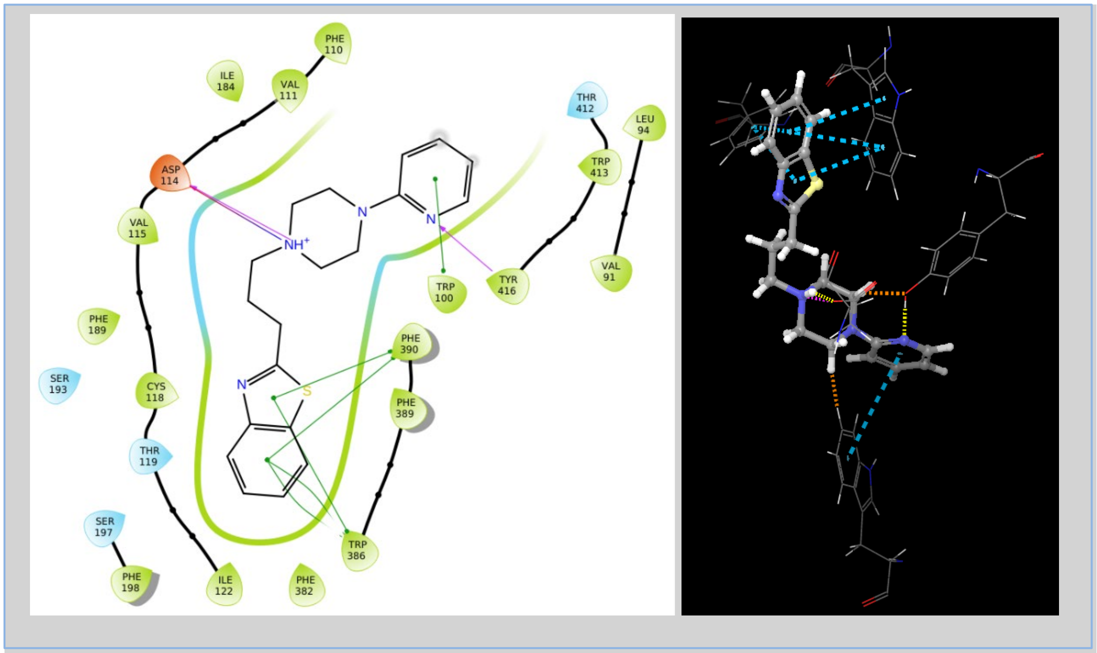

Table S3: CNS-MPO value and method of calculation for **16f**.[a]

CNS MPO Calculator		
**Property**	**Value**	**T0**
ClogP	3.8	0.600
ClogD	3.5	0.250
TPSA	32	0.600
MW	338.47	1.000
HBD	0	1.000
p*K*a	7.6	1.000
**CNS-MPO**		**4.5**

[a]ClogP: calculated logP water/octanol partitioning prediction; ClogD: predicted water/octanol partition value at pH 7.4; TPSA: topological polar surface area; MW: molecular weight; HBD: hydrogen bond donors; p*K*a: ionization constant of the most basic center. Values for ClogP, ClogD, and p*K*a were calculated using ChemAxon Marvin, version 18.5. Values for TPSA, MW, and HBD were calculated using ChemDraw, version 20.0. The CNS-MPO score was calculated according to the published protocol.[1]

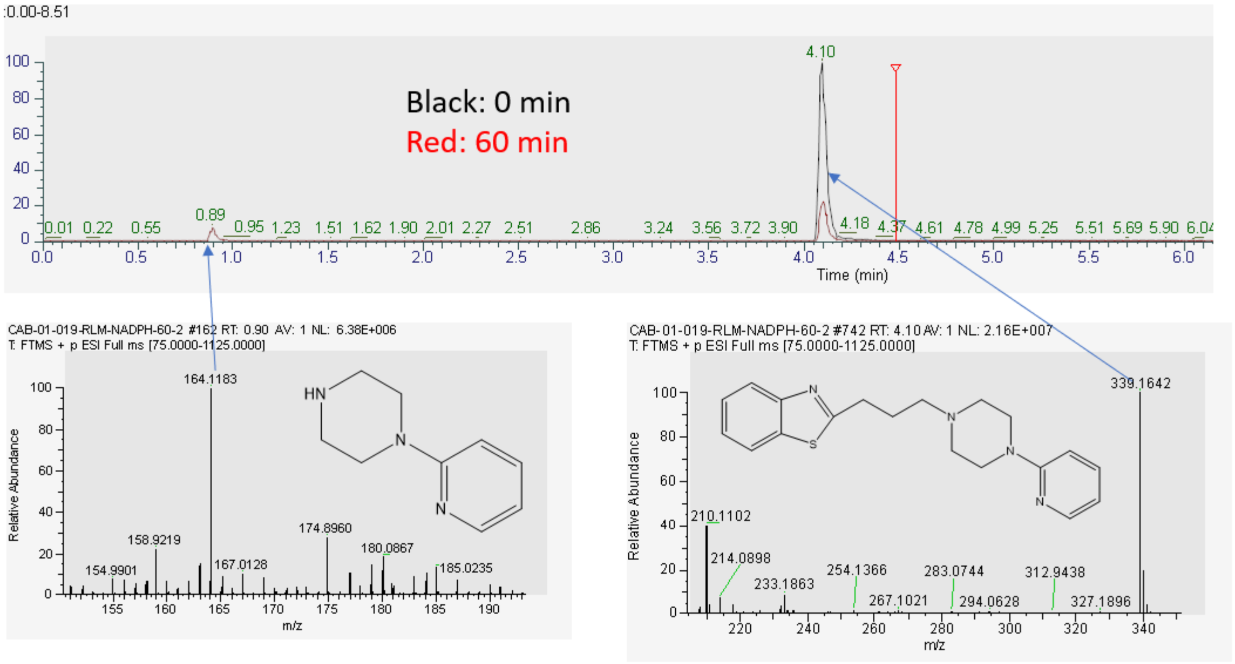

Figure S3. HPLC traces of **16f** 0 and 60 minutes after incubation with rat microsomes in the presence of NADPH (top). MS identification of the major dealkylated metabolite (left) and **16f** (right) are also shown.

Reference

1. Wager, T. T. Central nervous system multiparameter optimization desirability: application in drug discovery. *ACS Chem. Neurosci.* **2016,** 7, 767-775.
